# The glutaminase inhibitor telaglenastat enhances the antitumor activity of signal transduction inhibitors everolimus and cabozantinib in models of renal cell carcinoma

**DOI:** 10.1371/journal.pone.0259241

**Published:** 2021-11-03

**Authors:** Ethan Emberley, Alison Pan, Jason Chen, Rosalyn Dang, Matt Gross, Tony Huang, Weiqun Li, Andrew MacKinnon, Devansh Singh, Natalija Sotirovska, Susanne M. Steggerda, Tracy Wang, Francesco Parlati

**Affiliations:** Calithera Biosciences, Inc., South San Francisco, CA, United States of America; University of Nebraska Medical Center, UNITED STATES

## Abstract

Dysregulated metabolism is a hallmark of cancer that manifests through alterations in bioenergetic and biosynthetic pathways to enable tumor cell proliferation and survival. Tumor cells exhibit high rates of glycolysis, a phenomenon known as the Warburg effect, and an increase in glutamine consumption to support the tricarboxylic acid (TCA) cycle. Renal cell carcinoma (RCC) tumors express high levels of glutaminase (GLS), the enzyme required for the first step in metabolic conversion of glutamine to glutamate and the entry of glutamine into the TCA cycle. We found that RCC cells are highly dependent on glutamine for proliferation, and this dependence strongly correlated with sensitivity to telaglenstat (CB-839), an investigational, first-in-class, selective, orally bioavailable GLS inhibitor. Metabolic profiling of RCC cell lines treated with telaglenastat revealed a decrease in glutamine consumption, which was concomitant with a decrease in the production of glutamate and other glutamine-derived metabolites, consistent with GLS inhibition. Treatment of RCC cells with signal transduction inhibitors everolimus (mTOR inhibitor) or cabozantinib (VEGFR/MET/AXL inhibitor) in combination with telaglenastat resulted in decreased consumption of both glucose and glutamine and synergistic anti-proliferative effects. Treatment of mice bearing Caki-1 RCC xenograft tumors with cabozantinib plus telaglenastat resulted in reduced tumor growth compared to either agent alone. Enhanced anti-tumor activity was also observed with the combination of everolimus plus telaglenastat. Collectively, our results demonstrate potent, synergistic, anti-tumor activity of telaglenastat plus signal transduction inhibitors cabozantinib or everolimus via a mechanism involving dual inhibition of glucose and glutamine consumption.

## Introduction

Dysregulated metabolism is a hallmark of cancer, enabling tumor cells to sustain high rates of proliferation, even under unfavorable conditions of limited oxygen and essential nutrients [1, 2]. Glycolysis is enhanced in cancer cells as a means to support the biosynthesis of metabolic intermediates required for several pathways, including amino acid biosynthesis, the pentose phosphate pathway (PPP), and folate metabolism [3]. An increased reliance on aerobic fermentation—a phenomenon called the “Warburg Effect” whereby glucose is converted to lactate—results in reduced bioavailability of pyruvate for fueling the tricarboxylic acid (TCA) cycle [4, 5]. As a consequence, there is increased demand for an anapleurotic source of carbon to fuel the TCA cycle. This demand for anapleurotic carbon is satisfied by glutamine, which is converted to glutamate by the enzyme glutaminase (GLS) [6, 7]. Glutamate is further converted to α-ketoglutarate for entry into the TCA and the generation of energy and biomolecules, including nucleotides, fatty acids, and amino acids [5, 6, 8–10]. The heightened dependency on glutamine metabolism renders cancer cells sensitive to inhibition of GLS activity, making GLS an attractive target for cancer therapeutics.

In clear cell renal cell carcinoma (ccRCC), the most common histological variant of RCC, the Von Hippel-Lindau (VHL) tumor suppressor gene is silenced, which leads to activation of hypoxia inducible transcription factors, HIF-1α and HIF-2α. HIF-1α and HIF-2α drive metabolic reprogramming in RCC cells, which is characterized by enhanced glycolysis and reduced glucose oxidation, a compensatory increase in consumption of glutamine, increased flux of glutamine-derived carbons into the TCA cycle [11], and a reliance on glutamine for growth and proliferation [12–14]. Expression of HIF-2α has also been shown to promote reductive carboxylation of α-ketoglutarate in RCC cells, diverting the dependency of the TCA cycle away from glucose towards glutamine [13]. Compared to normal kidney, RCC cells express higher levels of the more active isoform of GLS known as GAC, which is consistent with increased glutamine utilization thereby creating a therapeutic vulnerability to GLS inhibition [12, 15–17].

In addition to the VHL/HIFα axis, many cancers, including RCC, have aberrant activation of signal transduction pathways dependent on the tyrosine kinases VEGFR, ALK, and MET. While these pathways are well known to drive tumor cell proliferation and survival, they also promote metabolic alterations via activation of the PI3K/AKT/mTOR pathway that result in increased aerobic glycolysis [18–20]. Inhibitors of VEGFR or mTOR downregulate glycolysis, limiting production of ATP and cellular building blocks [21–25]. Concordantly, inhibition of signal transduction pathways with receptor tyrosine kinase inhibitors, such as cabozantinib, or mTOR inhibitors, such as everolimus, have been associated with antitumor activity in a number of cancer types, including RCC [26, 27].

Telaglenastat (CB-839) is an investigational, first-in-class, selective, orally bioavailable, small molecule inhibitor of both splice variants (KGA and GAC) of glutaminase, currently under clinical investigation for the treatment of several cancers including RCC [28]. We hypothesized that targeting of both glutamine and glucose utilization pathways via dual inhibition of GLS and tyrosine kinase signalling pathways would lead to synergistic suppression of RCC tumor cell proliferation. Here we examined the anti-proliferative effects of telaglenastat, alone and in combination with signaling inhibitors, in RCC cell lines and tumor xenograft models in mice. We observed that dual inhibition of glutaminase and signal transduction pathways lead to synergistic anti-proliferative activity in vitro and enhanced anti-tumor activity in vivo.

## Materials and methods

### Cell lines

Cell lines, A-704, 786-O, Caki-1, A498, 769-P, Caki-2, ACHN and G401 were obtained from ATCC; RCC-JW, RCC-JF, RCC-MF, RCC-GH, RCC-FG1, WT-CLS1, RCC-ER and RCC-FG2 were obtained from Cell Line Service (CLS); CAL-54 and BFTC-909 were obtained from the German Collection of Microorganisms and Cell Cultures (DSMZ); VMRC-RCZ, VMRC-RCW, JMU-RTK-2, KMRC-20, KMRC-1 and KMRC-3 were obtained from JP Cell Bank and TUHR10TKB, OS-RC-2 and RCC-10RGB were obtained from Riken. Cells were maintained in RPMI-1640 media supplemented with 2 mM glutamine and 10% fetal bovine serum. Cell lines were purchased from vendors that perform routine authentication of their human cell lines and were used at low passage number.

### Cell viability assays

For viability assays, cell lines were treated with vehicle (DMSO), telaglenastat, everolimus, cabozantinib, or the indicated combinations for 72 hours in triplicate wells and analyzed for proliferation using CellTiter-Glo (CTG; Promega). For all cell lines, the results presented are representative of at least two independent experiments. IC_50_ values were calculated using a four parameter curve fit (GraphPad Prism). Relative cell loss or proliferation in the presence of 1 μmol/L telaglenastat or in glutamine-free media was determined by comparing the CTG signals measured at time (*t*) = 72 hours under experimental conditions (CTG_exp_72_) with both the CTG signal at *t* = 72 hours for vehicle (0.5% DMSO) treated cells (CTG_DMSO_72_) and the CTG signal measured at *t* = 0, the time of telaglenastat addition or glutamine withdrawal (CTG_0_), using the following equations: % cell loss (when CTG_exp_72_ < CTG_0_) = 100 × (CTG_exp_72_ − CTG_0_)/CTG_0_; % cell proliferation (when CTG_exp_72_ > CTG_0_) = 100 × (CTG_exp_72_ − CTG_0_)/(CTG_DMSO_72_ − CTG_0_). Combination indices were calculated by the method of Chou–Talalay using Calcusyn software [29].

### Metabolite analysis

Glutamine, glutamate, malate, citrate, and glutathione were measured in ACHN and TUHR10TKB cell lines. Cells were homogenized in methanol:water (80:20) containing 10 μmol/L of the following internal standards: L-glutamine-^15^N_2_, ^13^C_5_; L-glutamic acid-^13^C_5_, ^15^N; L-malic acid-^13^C_4_; and glutathione-(glycine-^13^C_2_, ^15^N). Metabolite levels were analyzed by liquid chromatography-tandem mass spectrometry (LC/MS-MS) using a SCIEX API4000 mass spectrometer (Applied Biosystems), and statistical analyses were conducted using t tests.

### Cell culture media metabolites and seahorse experiments

For experiments quantifying metabolite consumption or production in tissue culture media, cells were incubated in DMEM with 5 mmol/L glucose and 0.5 mmol/L glutamine (no serum) for 24 hours. Media concentrations of glucose, lactate, glutamine, and glutamate were quantified using the YSI 2900 Biochemistry Analyzer (YSI Life Sciences).

To quantify rates of oxygen consumption and extracellular acidification, cells were seeded (20,000 cells/well) in RPMI medium containing 5% FBS on XF96 V3 PET plates (Seahorse Biosciences). After the cells were attached overnight, the medium was exchanged with DMEM (5 mmol/L glucose with or without 0.5 mmol/L glutamine, no FBS, no bicarbonate) and the plates were immediately loaded onto the Seahorse Biosciences XF96 Bioanalyzer for quantification of oxygen consumption rates (OCR). To determine effects on extracellular acidification rate (ECAR), compounds were added sequentially (telaglenastat and/or everolimus [Selleck Chemicals, #S1120], cabozantinib [Selleck Chemicals, #S1119], DMSO, or the indicated combinations, 80 mM glucose, 9 μM oligomycin, and 500 mM 2-deoxyglucose) for 24 hours.

Statistical analyses were conducted using Brown-Forsythe and Welch 1-way ANOVA with Dunnett’s test for multiple comparisons.

### Immunohistochemical staining

RCC tumors were purchased from Indivumed (Hamburg, Germany). Immunohistochemical staining was performed at Covance Laboratories Inc. (Greenfield, USA), as previously described [30]. Tumors were fixed in 10% neutral-buffered formalin, embedded in paraffin, and cut into 4 μm sections, which were then dried, deparaffinized, and rehydrated. Heat-induced antigen retrieval was performed for 10 minutes (Antigen Retrieval Solution, Leica, AR9661). Endogenous peroxidase activity was quenched with peroxidase block. Sections were incubated with primary antibody (rabbit anti-human GLS, Abcam, ab156876, 1:200 dilution) for 15 minutes at room temperature. Immunohistochemical reactions were visualized using Dako Rabbit Envision+HRP with DAB+ for use with rabbit primary antibodies (K4011, Dako). Sections were counterstained with hematoxylin.

### Western blot analysis

Lysates were prepared from tumor cell line pellets by sonication in cell homogenization buffer (50 mM Tris-Acetate pH 8.6, 150 mM K2HPO4, 0.25 mM EDTA, 1 mM DTT, 1X complete protease inhibitors) using a Bioruptor sonication device (Diagenode) for 5 minutes at 4°C on high power (30 seconds on/30 seconds off per 1 minute cycle). The homogenates were gel filtered on spin columns equilibrated in cell homogenization buffer supplemented with 0.01% Triton X-100. Gel-filtered lysates were quantified for total protein (Pierce), snap frozen in liquid nitrogen, and stored at -80°C. Lysates (20 μg/lane) were denatured by boiling in SDS-sample buffer, resolved on Tris-acetate gels together with Novex sharp pre-stained molecular weight standards (Life Technologies), and transferred to nitrocellulose membranes. Nitrocellulose-immobilized proteins were probed with the following antibodies, all purchased from Cell Signaling Technology, unless indicated otherwise: phospho-S6 (#5364), S6 (#2217), phospho-AKT (Ser473, #4060), AKT (#4691), phospho-Erk1/Erk2 (Tyr187/Tyr204, #5726S), Erk (#9102), phospho-4E-BP1 (Ser65, #9456), and 4E-BP1 (#9644), and β-actin (1:10,000; A5441, Sigma-Aldrich) followed by horseradish peroxidase-coupled anti-rabbit or anti-mouse antibodies (1:5,000; NA934V and NA931V, GE Healthcare). Bands were revealed by chemiluminescence (Thermo Scientific) and images were captured with a FluorChem HD2 system (Protein Simple).

### Animals

Protocols for animal use were reviewed and approved by Calithera’s Institutional Animal Care and Use Committee (IACUC; protocol #CAL-003). Animals were cared for as described in *Guide for the Care and Use of Laboratory Animals* (National Research Council, Washington, DC, National Academy Press). Mice were housed within Calithera’s onsite vivarium where the environmental conditions were as follows: between 68° and 79°F, 30% to 70% humidity, and 12-hour light-dark cycles. Mice were group housed in polycarbonate cages with appropriate bedding to keep the animals clean and dry. Cages were placed in ventilated racks providing HEPA filtered air. Water and pelleted rodent food were provided ad libitum. Environmental enrichment items (nestlets or puff squares) were provided at each cage change. Immunocompromised animals were handled in a biosafety cabinet.

Procedures were followed to ensure that discomfort and injury to animals were limited to that which was unavoidable to conduct the studies. Mice were observed daily and tumors were measured with calipers 3 to 4 times per week. Mice were euthanized if tumors reached 2000 mm^3^. Per IACUC protocol, mice were to be euthanized if any of the following clinical signs were observed: body weight loss of 20% compared to vehicle-matched controls, diarrhea more than 48 hours in duration, self-induced trauma, bleeding from any orifice, neurological signs incompatible with maintenance of normal life functions (e.g., inability to eat, drink, ambulate), hyper- or hypothermia for more than 24 hours, respiratory difficulties, nonresponsiveness to stimuli, moribund condition, or tumors with discharge. None of these clinical signs were observed in any animals on study and no mice died before they could be euthanized. Mice were euthanized by carbon dioxide asphyxiation followed by exsanguination or cervical dislocation.

### *In vivo* studies

Mice (female SCID/beige) were purchased from Charles River Laboratories. N = 160 mice were used for the studies. Mice were implanted subcutaneously with 2.5x10^6^ Caki-1 RCC cells plus Matrigel per mouse. When tumors reached an average of ~400 mm^3^, mice in the everolimus combination studies were randomized into the following 4 treatment groups: (i) vehicle, (ii) telaglenastat at 200 mg/kg and dosed orally twice daily (BID), (iii) everolimus at 1 mg/kg dosed orally once daily (QD), and (iv) telaglenastat and everolimus (Selleck Chemicals, #S1120) at 200 mg/kg and 1 mg/kg, respectively. For the cabozantinib (and other TKI combination studies), mice were randomized into the following 4 treatment groups: (i) vehicle, (ii) telaglenastat at 200 mg/kg and dosed orally BID, (iii) cabozantinib at 1 mg/kg dosed orally QD, and (iv) telaglenastat and cabozantinib (Selleck Chemicals, #S1119). Sunitinib (Selleck Chemicals, #51042) was dosed orally at 20mg/kg QD and axitinib (MedChemExpress, #HY-10065) at 25mg/kg QD. Statistical analyses were conducted using Ordinary ANOVA with Tukey’s multiple comparison tests.

## Results

### RCC cell lines are sensitive to glutaminase inhibition with telaglenastat

**Fig 1:**
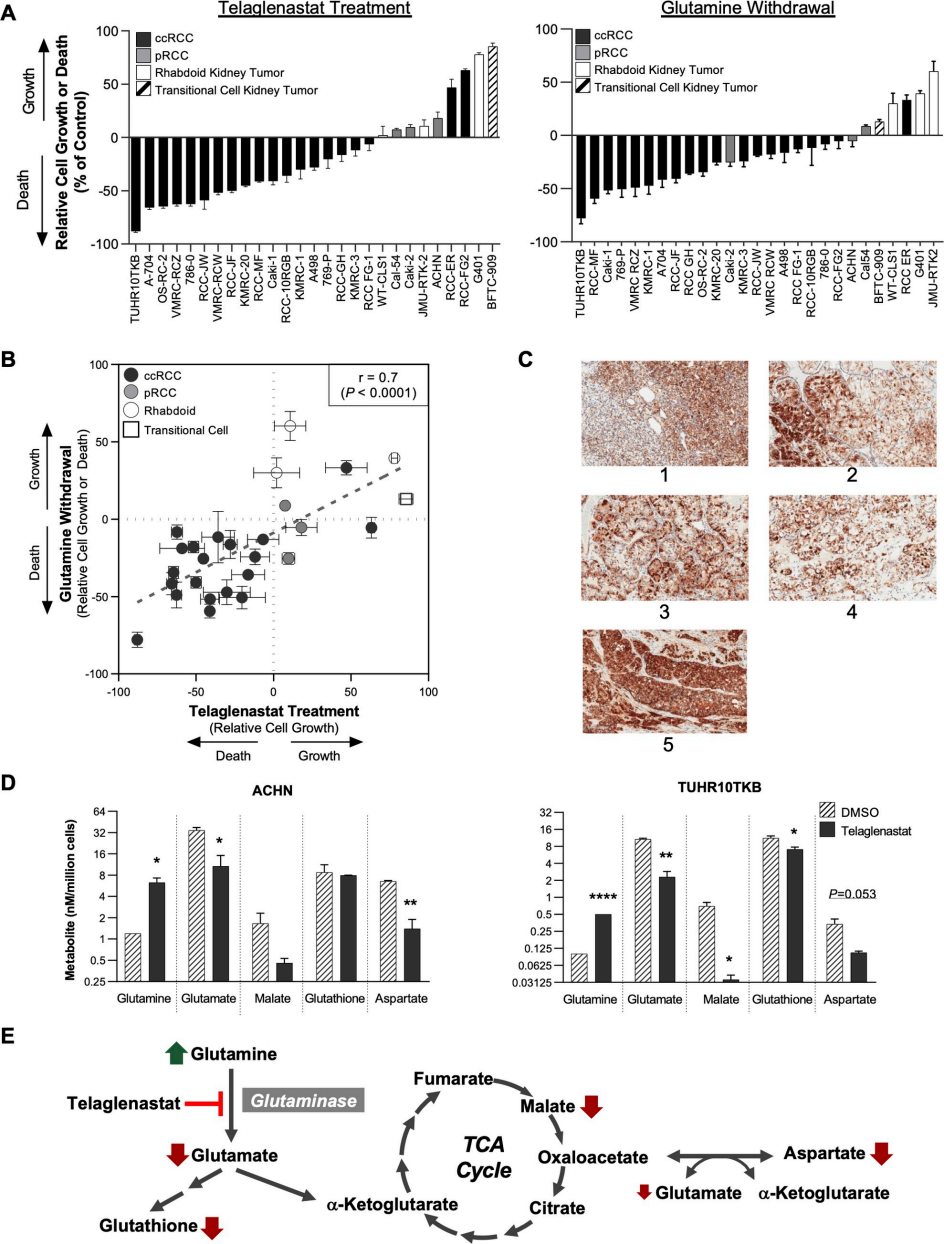
Sensitivity of ccRCC and pRCC cells to telaglenastat correlates with glutamine dependency and is associated with reduced metabolites downstream of glutamine. (A) Relative cell growth or cell death across a panel of kidney cancer cell lines following incubation with telaglenastat (1 μM; left panel) or glutamine withdrawal (right panel) for 72 hours. (B) Correlation between telaglenastat sensitivity (1 μM) and response to glutamine withdrawal at 72 hours. Each data point on the bivariate plot depicts an individual cell line. (C) Immunohistochemical staining of glutaminase in primary ccRCC tumors. (D) Intracellular metabolite levels of glutamine and its downstream metabolites following 1 μM telaglenastat treatment for 4 hours. Graphs represent two independent experiments (left panel: ACHN cell line; right panel: TUHR10TKB cell line) performed in duplicate. (E) Schematic representation of glutamine metabolism showing experimentally observed changes to levels of glutamine-derived metabolites following treatment with telaglenastat. For (A) and (B), data represent result of 2 or 3 independent experiments, each performed in triplicate. Error bars represent standard error of the mean. For (D), statistical analyses were conducted using t tests: *P ≤ 0.05; **P ≤ 0.01; ***P ≤ 0.001.

To determine the extent to which RCC cell lines are dependent on glutamine, we first evaluated the effect of glutamine withdrawal in a panel of 27 kidney tumor cell lines, including 20 ccRCC, 3 papillary RCC (pRCC), 3 rhabdoid tumor of the kidney, and 1 kidney transitional cell carcinoma cell line. RCC cell lines displayed a cytotoxic or strongly cytostatic response to glutamine withdrawal in 18 of 20 ccRCC cell lines and in all 3 pRCC cell lines (**Fig 1A, right panel; S1 Table**). Rhabdoid kidney and transitional kidney cell lines were largely insensitive to glutamine withdrawal (**Fig 1A; S1 Table**). RCC cell lines were next evaluated for the ability to proliferate in the presence of 1 uM telaglenastat (**Fig 1A, left panel**). Treatment with telaglenastat was found to induce cell death in 16 out of 20 ccRCC cell lines, with reductions in cell number ranging from –88% to –9% relative to the start of drug treatment (**Fig 1A**). Teleglenastat also showed pronounced cytostatic effects in 2 additional ccRCC cell lines and 3 pRCC cell lines, with cell numbers ranging from 3% to 17% of the control-treated cultures. Using the same cell line panel, the anti-proliferative effect of glutaminase inhibition was assessed using a dose titration of telaglenastat (from 0.15 nM to 1 μM) to determine an EC_50_ after three days of growth. Generally consistent with the effects of glutamine withdrawal, 18 out of 20 ccRCC cell lines and all 3 pRCC were sensitive to telaglenastat treatment, whereas the kidney rhabdoid and transitional cell carcinoma cell lines were resistant (**S1 and S2 Figs**).

Sensitivity of RCC cells to telaglenastat strongly correlated with dependence on glutamine, indicating that RCC cells use glutamine to support glutaminolysis through the activity of GLS (r = 0.7, *P* < 0.0001; **Fig 1B**). In contrast, most of the non-RCC cell lines (i.e., rhabdoid and transitional cell kidney tumors) were less sensitive to telaglenastat, showing only partial cytostatic effects or complete resistance. Therefore, RCC cells appear to be highly sensitive to inhibition of GLS compared to non-RCC kidney tumor cells.

Given the dependency on GLS in RCC cell lines, we looked at GLS expression in RCC tumors. Across all tumor types in The Cancer Genome Atlas (TCGA) database [31–33], RCC had the highest median expression level of GLS; within RCC, the highest levels were found in pRCC, followed by ccRCC (**S3 and S4 Figs**). Immunohistochemical staining in 5 primary human ccRCC tumors revealed high GLS expression (**Fig 1C**). These data suggest that, like RCC cell lines, RCC tumors have an elevated dependency on GLS and glutamine metabolism.

To confirm the anti-proliferative effects of telaglenastat resulted from GLS pathway inhibition, we measured changes in the levels of intracellular metabolites downstream and upstream of GLS in cells treated for 4 hours with telaglenastat. Telaglenastat treatment led to a mean ~5-fold increase in glutamine levels, while intracellular levels of glutamate, glutathione, malate, and aspartate showed marked decreases compared with control-treated cells (**Fig 1D**). These data are consistent with an on-target mechanism of action of telaglenastat in RCC cell lines (**Fig 1E**) [28].

### Telaglenastat synergizes with everolimus to inhibit proliferation of RCC cells

**Fig 2:**
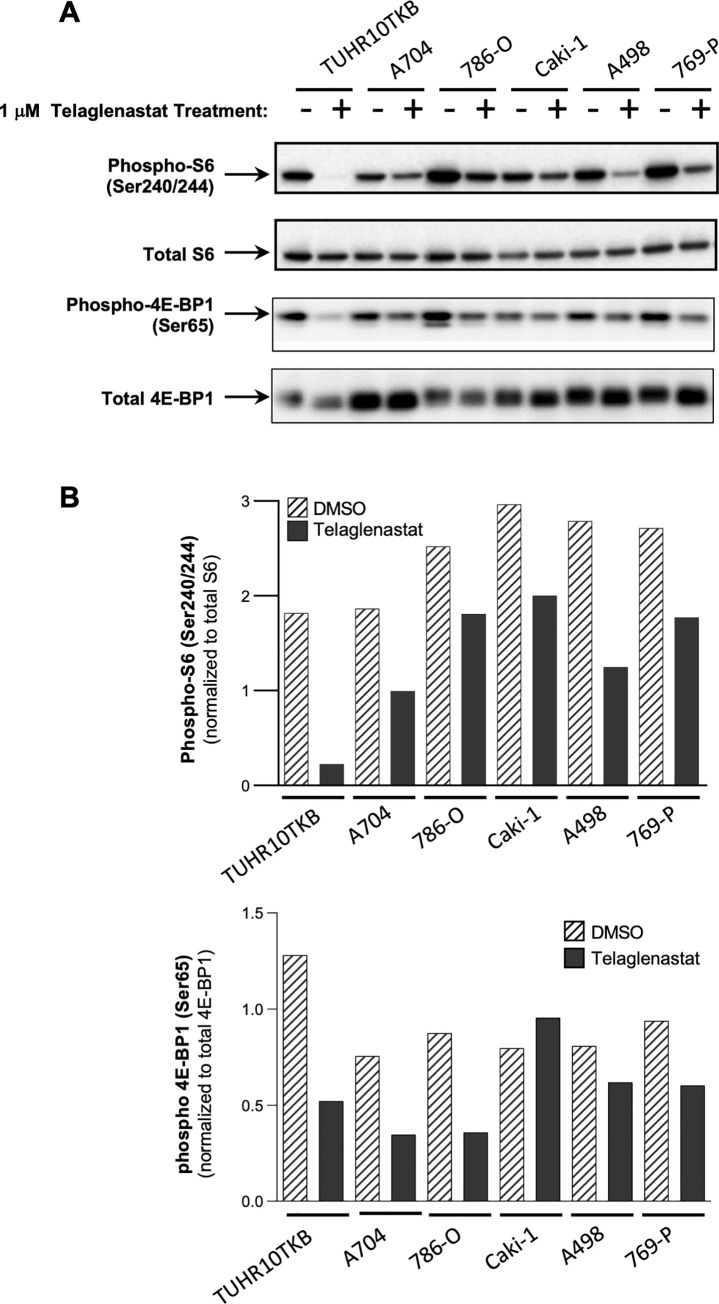
Telaglenastat inhibits the mTORC1 pathway in RCC cells. (A) Western blot of phospho-S6, total S6, phospho-4E-BP1 and total 4E-BP1 in RCC cell lines after 24 hours of telaglenastat treatment (1 μM) or DMSO control. (B) Relative phospho-S6 and phospho-4E-BP1 levels normalized to total S6 and 4E-BP1, respectively, quantified by densitometry. Representative blots of at least two independent experiments are shown.

Previous studies have shown amino acid starvation suppresses activation of mammalian target of rapamycin (mTOR) pathways, which in turn, enables the autophagy response to nutrient deprivation [34, 35]. To determine whether telaglenastat could suppress mTOR pathway activity in RCC, we measured phosphorylation of S6 and 4E-BP1, markers of mTOR activation, across 6 ccRCC cell lines. Incubation of cells in the presence of telaglenastat resulted in decreased phosphorylation of S6 and 4E-BP1, compared with the DMSO control (**Fig 2**). These findings demonstrate that telaglenastat may indirectly inhibit mTOR signaling by depriving cells of metabolites produced by the breakdown of glutamine by GLS, consistent with previous findings [36].

**Fig 3:**
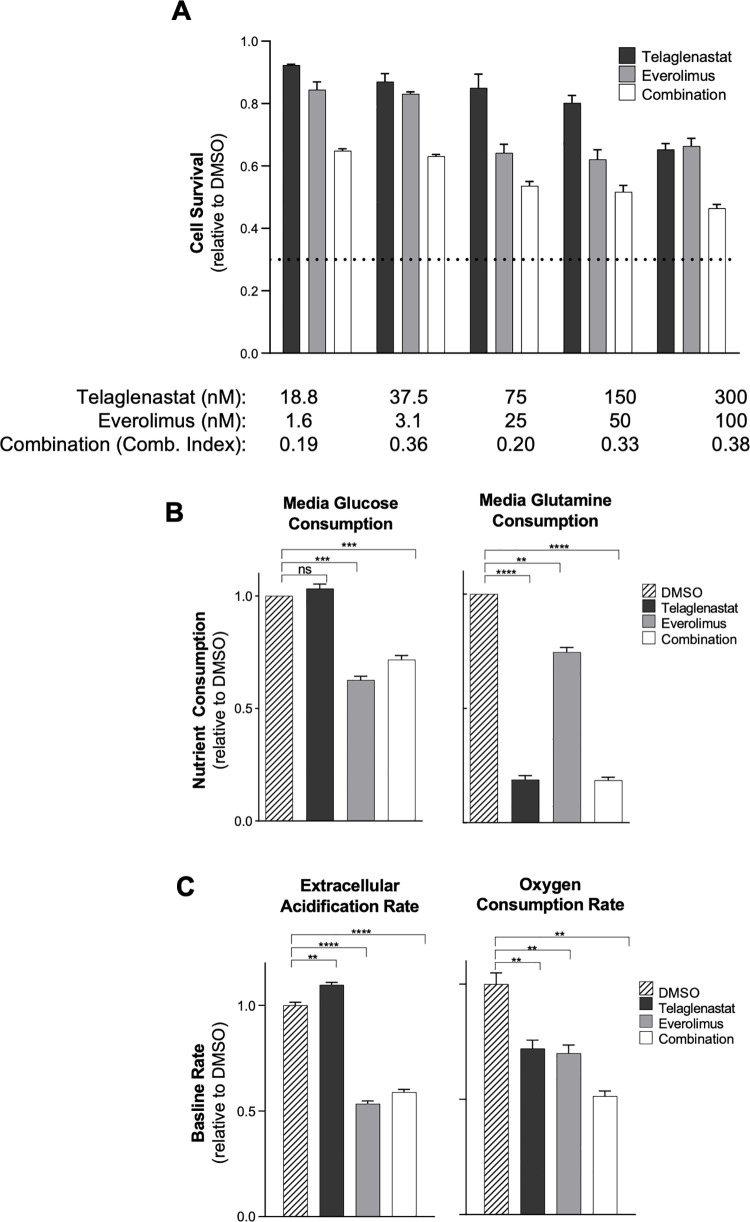
Synergistic anti-proliferative activity and disruption of glutamine and glucose metabolism by telaglenastat and everolimus in RCC cells. (A) Viability of ACHN cells treated with telaglenastat, everolimus, or a combination of both inhibitors for 72 hours. Dotted line indicates the baseline CellTiter-Glo signal at the time of compound addition. (B) Measurements of glucose or glutamine consumption from media of ACHN cells treated with 75 nM telaglenastat, 25 nM everolimus, or the combination of both inhibitors for 24 hours. (C) Measurements of ECAR and OCR in ACHN cell cultures. ACHN cells were treated with DMSO, telaglenastat (75 nM), everolimus (25 nM), or the combination of both inhibitors for 24 hours and analyzed using the Seahorse Metabolic Analyzer. All experiments were performed in triplicate or quadruplicate. Error bars represent standard error of the mean. Statistical analyses were conducted using Brown-Forsythe and Welch 1-way ANOVA with Dunnett’s test for multiple comparisons: ***P ≤ 0.001; ****P ≤ 0.0001; ns = nonsignificant.

Everolimus is a potent mTOR inhibitor that is approved for the treatment of advanced/metastatic RCC [37]. Metabolic effects of inhibiting mTOR with everolimus in patients include hyperglycemia, attributed to suppression of key glycolytic enzymes and the pentose phosphate pathway [37, 38]. Given our observations of telaglenastat’s inhibitory effect on mTOR signaling, we explored the combined antiproliferative effects of telaglenastat with everolimus in RCC cells. ACHN cells were incubated for 72 hours with a range of concentrations of telaglenastat, everolimus, or the combination. Dose-dependent decreases in cell survival were observed with both single agent treatments, and the combination of telaglenastat with everolimus showed synergistic antiproliferative effects, with combination indices [29] ranging from 0.19 to 0.38 (**Fig 3A**). Similar results were observed in TUHR10TKB cells (**S5 Fig**).

To evaluate the mechanism of the antiproliferative effect of telaglenastat and everolimus on RCC cells, we first measured consumption of glucose and glutamine from cell culture media of ACHN cells treated for 24 hours with telaglenastat, everolimus, or the combination (**Fig 3B**). As expected, telaglenastat reduced glutamine consumption by more than 80% compared to the vehicle control, but did not alter glucose consumption. Conversely, everolimus reduced glucose consumption to 63% of the control. Combination of telaglenastat with everolimus inhibited both glucose and glutamine consumption by ACHN RCC cells.

Extracellular acidification rate (ECAR) and oxygen consumption rates (OCR) are frequently used as measures of glycolysis and mitochondrial respiration, respectively [39]. Consistent with the suppressive effects of everolimus on glycolysis, both ECAR and OCR decreased with everolimus treatment in all 8 RCC cell lines tested (**Fig 3C**, **S6 Fig**). Telaglenastat, on the other hand, primarily reduced OCR, but not ECAR, demonstrating its specificity in inhibiting GLS and entry of glutamate into the TCA cycle. A combined inhibitory effect was observed with telaglenastat plus everolimus in OCR, but not ECAR. The dual effect of the combination of telaglenastat and everolimus on both ECAR and OCR may explain the anti-proliferative synergy we observed and is consistent with other groups’ findings showing enhanced activity of mTOR inhibtiors when combined with GLS inhibition [36, 40, 41].

### Telaglenastat synergizes with cabozantinib to inhibit proliferation of RCC cells

**Fig 4:**
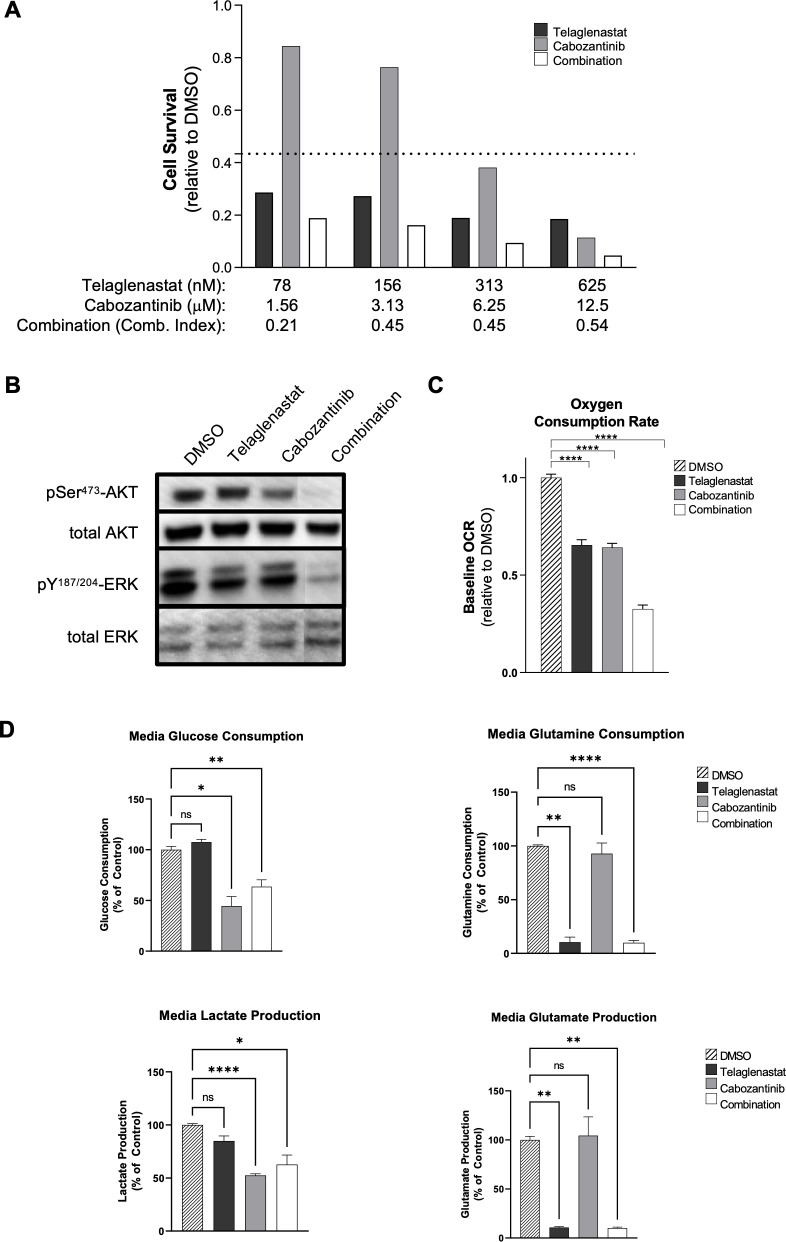
Synergistic anti-proliferative activity of telaglenastat and cabozantinib in ccRCC cells. (A) Viability of Caki-1 cells treated with telaglenastat, cabozantinib, or a combination of both inhibitors for 72 hours. The dashed line indicates the baseline CellTiter-Glo signal at the time of compound addition. (B) Western blots of total Akt and phospho-Akt and total Erk and phospho-Erk. A representative blot of at least two independent experiments is shown. (C) Measurement of OCR in Caki-1 cells treated for 24 hours with the indicated compounds. (D) Glucose and glutamine consumption and lactate and glutamate production, collected in media from Caki-1 cells after 24 hours of treatment with the indicated compounds. For parts (B)-(D), Caki-1 cells were treated with DMSO, telaglenastat (1 μM), cabozantinib (6 μM) or the combination of both inhibitors for 24 hours. Sample sizes were n = 5 or 6 for each condition. Error bars represent standard deviations. Statistical analyses were conducted using Brown-Forsythe and Welch 1-way ANOVA with Dunnett’s test for multiple comparisons: *P ≤ 0.05; **P ≤ 0.01; ****P ≤ 0.0001.

Cabozantinib is an orally administered tyrosine kinase inhibitor approved for treatment of advanced/metastatic RCC. Cabozantinib inhibits growth factor receptors, VEGFR, MET, and AXL, and has downstream effects on the PI3K-AKT-mTOR pathway, leading to decreased glucose utilization [25]. To evaluate the combined effect of inhibiting glutamine and glucose metabolic pathways in RCC cells, we measured cell survival of Caki-1 cells after 72 hours of treatment with telaglenastat, cabozantinib, or both agents in combination. While telaglenastat and cabozantinib each exhibited dose-dependent single agent reduction in cell survival, the combination showed synergistic activity, with combination indices ranging from 0.25 to 0.54 (**Fig 4A**).

Next, to understand the effect of telaglenastat and cabozantinib on cell signaling, we investigated activation of Akt and Erk, which lie downstream of c-Met. Western blot analysis showed reduced phospho-Akt in the presence of cabozantinib and reduced phospho-Erk in the presence of either cabozantinib or telaglenastat, compared with the DMSO control (**Fig 4B**). A greater decrease in phosphorylation was observed with both phospho-Akt and phospho-Erk in the presence of both agents, further supporting the combined effect of telaglenastat with cabozantinib on growth factor signaling pathways.

Findings of OCR inhibition in RCC cells with telaglenastat and cabozantinib were similar to those of telaglenastat plus everolimus, with the combination exhibiting a greater decrease in OCR than either agent alone (**Fig 4C**). This decrease in OCR may be a result of reduced glucose and glutamine consumption. To that end, we evaluated the downstream metabolic effects of telaglenastat plus cabozantinib on glucose and glutamine consumption by Caki-1 cells. Cabozantinib inhibited glucose consumption and lactate production, while telaglenastat inhibited glutamine consumption and glutamate production (**Fig 4D**). The anti-proliferative synergy observed with the combination of telaglenastat plus cabozantinib may be explained by the dual blockade of both glucose and glutamine consumption.

### RCC xenografts: Telaglenastat + everolimus, cabozantinib, sunitinib or axitinib

**Fig 5:**
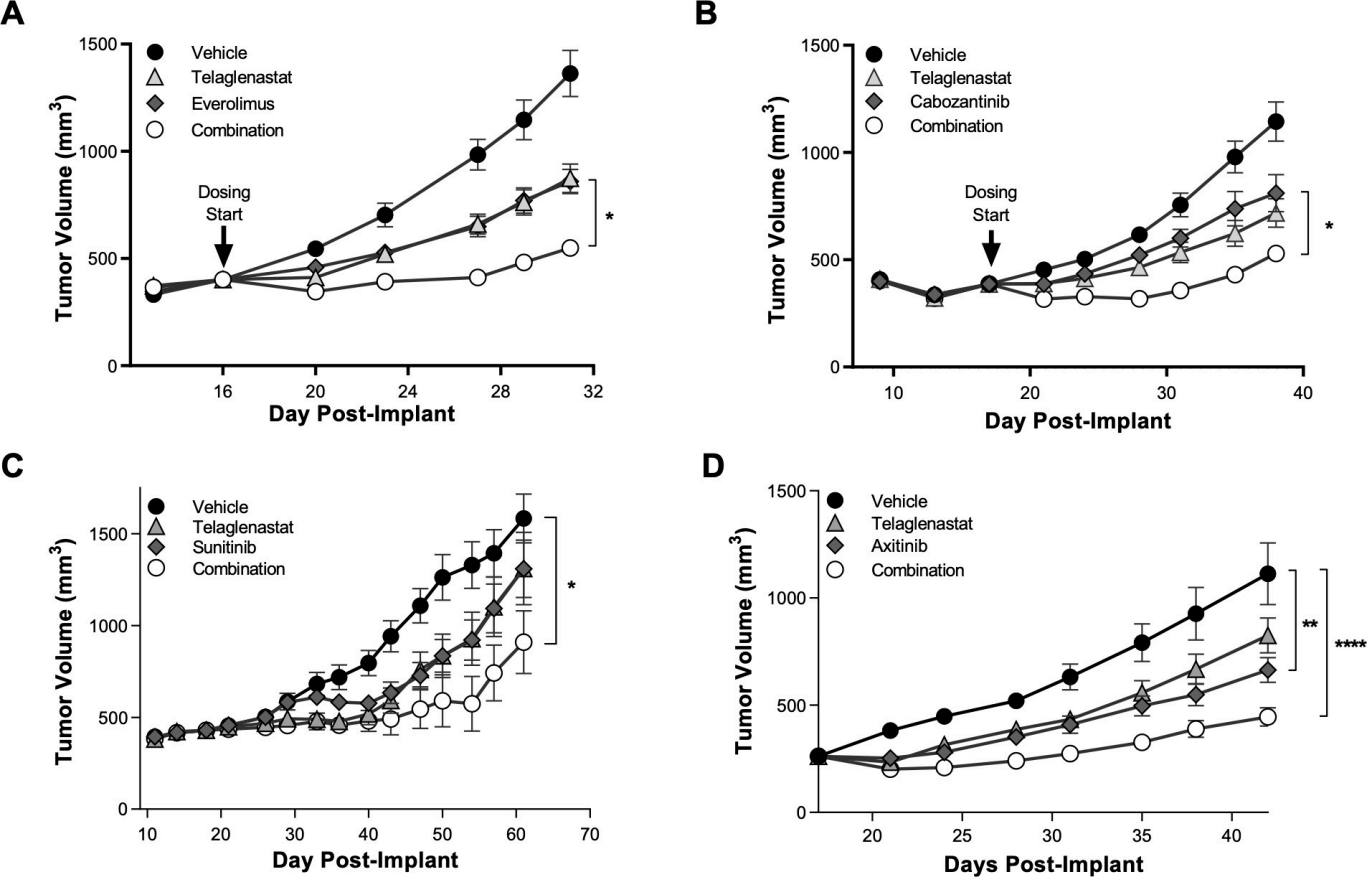
Telaglenastat enhances the antitumor activity of mTOR, VEGFR, or receptor tyrosine kinase inhibitors in vivo. Tumor volumes were measured in mice implanted subcutaneously with Caki-1 RCC cells and treated with either vehicle, telaglenastat (200 mg/kg, dosed orally BID), or (A) everolimus (1 mg/kg, dosed orally QD), (B) cabozantinib (1 mg/kg dosed orally QD), (C) sunitinib (20 mg/kg dosed orally QD), or (D) axitinib (25 mg/kg dosed orally QD) alone or combinations of telaglenastat with each. Statistical analyses were conducted using Ordinary 1-way ANOVA with Tukey’s multiple comparison tests: *P ≤ 0.05; **P ≤ 0.01; ****P ≤ 0.0001.

Given the pronounced synergy observed when combining telaglenastat with everolimus or cabozantinib in vitro, we next tested telaglenastat for anti-tumor activity in a Caki-1 mouse xenograft model of ccRCC. Treatment of tumor-bearing mice with telaglenastat led to a slower rate of tumor growth than mice treated with the vehicle control (**Fig 5**). Similar decreases in the rate of tumor growth were observed with single agent treatments of everolimus, cabozantinib, sunitinib, or axitinib. Antitumor activity was significantly greater when telaglenastat was combined with everolimus, cabozantinib, sunitinib, or axitinib (*P* < 0.05; **Fig 5**). All combinations were well tolerated in mice, with no notable changes in body weights compared with vehicle control (**S7 Fig**).

## Discussion

The rationale for glutaminase inhibition as a therapeutic strategy for RCC is based on extensive studies of the Warburg effect and glutamine addiction in RCC cells. The Warburg effect is characterized by enhanced glucose uptake and glycolytic flux that enables pyruvate to bypass the TCA cycle and undergo reduction to lactate in proliferating cancer cells [4, 5]. In many cancers, including RCC, anaplerosis of the TCA cycle is sustained via the conversion of glutamine to α-ketoglutarate, the first step of which is mediated by GLS [6]. Several lines of evidence have pointed to RCC being a metabolically active tumor. First, fluorodeoxyglucose-positron emission tomography (FDG-PET) studies have shown that metastatic RCC lesions are FDG avid, meaning RCC tumors consume high amounts of glucose, with high levels of FDG uptake correlating with poor prognosis [42–48]. In addition, in vivo isotope tracing experiments in patients with ccRCC have shown high flux of ^13^C-glucose into glycolysis and high production of lactate. These findings are accompanied by low glucose flux into the TCA cycle and low levels of aspartate and glutamate in tumors compared to adjacent normal kidney tissue [49]. The tumor-specific enrichment in glycolytic intermediates coincides with decreases in glucose-derived TCA cycle intermediates, consistent with the Warburg effect. Diversion of glucose-derived metabolites away from the TCA cycle in ccRCC tumors thereby creates a dependency on alternative pathways for sustaining the levels of TCA cycle intermediates.

Glutamine utilization in tumors has been observed in both clinical and preclinical studies. PET scanning of cancer patients injected intravenously with the radiolabeled glutamine analog, fluoro-glutamine (^18^F-FGln), showed high uptake into tumors harboring mutations in metabolic genes, including an RCC patient with a germline mutation in the *SDHB* gene, which encodes a subunit of succinate dehydrogenase [50]. In another study, ^18^F-FGln uptake was observed across multiple tumor types, including patients with RCC and non-small cell lung cancer (NSCLC) who, upon treatment with telaglenastat, decreased their uptake of ^18^F-FGln [51]. Several nonclinical studies have also demonstrated elevated glutamine utilization in RCC tumors [16, 17, 52–55]. These studies characterize glutamine as a required substrate for generating glutathione to maintain redox balance [16, 53], for entering the TCA cycle [54], and for driving lipogenesis via reductive carboxylation [13, 55]. More recently, glutamine-derived aspartate was identified as a precursor for nucleotide biosynthesis [12, 56]. Taken together, these data indicate that glutamine fuels diverse metabolic pathways essential for cancer cells in ccRCC and other tumor types, both *in vitro* and in cancer patients.

Glutamine dependency is increased under conditions of hypoxia or upon activation of HIF transcription factors that upregulate enzymes that drive reductive carboxylation of glutamine in RCC [57]. VHL loss drives the metabolic phenotype in RCC by creating a pseudo-hypoxic state. Activation of HIF in response to loss of VHL leads to upregulated expression of glucose transporters (i.e., GLUT) and glycolytic enzymes (e.g., HK1, HK2, GPI, ALDOA/C, TPI1, GAPDH, PGK1, PGM, ENO1, and PKM2), and enzymes in the PPP (G6PD, TKT, TKTL2) [58]. Increased lactic acid production has also been observed, confirming that the Warburg effect in RCC is driven by VHL loss [58]. Although there is little evidence that HIF increases expression of glutaminolysis genes, several studies have shown that VHL loss increases expression of PKD1, thereby decreasing the activity of PDH, leading to lower utilization of glucose in the TCA cycle and an increased dependence on glutaminolysis for anapleurosis [58]. Increased expression of HIF may explain increased rates of glutaminolysis to fuel the TCA cycle in VHL-deleted tumors. These observations motivated our efforts to test the efficacy of glutaminase inhibition in RCC cells [13, 14].

In this study, we showed that RCC cell lines are sensitive to glutamine withdrawal and that this sensitivity correlated with sensitivity to glutaminase inhibition with telaglenastat. Telaglenastat had single agent anti-proliferative activity in RCC cell lines, consistent with previously published reports showing sensitivity of RCC cell lines to glutaminase inhibition [12, 13, 16]. Telaglenastat was more potent in ccRCC compared to pRCC cell lines, with the caveat that only 3 pRCC cell lines were available for testing. An on-target mechanism of action of telaglenastat in RCC cell lines is supported by our results that teleglenastat treatment resulted in reduced levels of glutamate and its direct downstream metabolites. Telaglenastat treatment of RCC cells decreased production of glutathione, the TCA cycle intermediate malate, and the amino acid asparate, confiming previous observations that glutamine fuels glutathione production and TCA cycle function [13, 28]. In addition, we showed that treatment with telaglenastat resulted in decreased glutamine consumption in the ACHN and Caki-1 cell lines. OCR, a measure of TCA cycle function, was decreased in all RCC cell lines treated with telaglenastat, indicating that this finding is generally observed in RCC cell lines. In vivo, telaglenastat controlled tumor growth in Caki-1 cells, consistent with observations of BPTES-based GLS inhibition in mouse models of RCC [12, 17].

After establishing the antitumor activity of telaglenastat as a single agent in ccRCC cell lines, we determined whether the effect of telaglenastat could be enhanced by combination with agents that target other metabolic pathways. Glucose consumption and lactate production are inhibited in RCC cells by everolimus and other mTOR inhibitors, thus representing a second targetable metabolic pathway [21]. Telaglenastat was previously shown to decrease mTOR activity in triple-negative breast cancer cell lines, demonstrating synergy with mTOR inhibition in the latter [36]. Similarly, we found that telaglenastat downregulated the PI3K/mTOR pathway in RCC cells and that the combination of everolimus with telaglenastat had synergistic antitumor effects in these cells. ECAR, a surrogate marker for glycolysis, was decreased in all 8 RCC cell lines tested following treatment with everolimus alone or in combination with telaglenastat. Exploration of the mechanism of action of the drug combination in ACHN cells showed decreases in both glucose and glutamine consumption, leading to decreased rates of ECAR and OCR in vitro. When combined in vivo, we observed enhanced antitumor activity in a Caki-1 xenograft model of RCC. Other groups have reported that mTOR inhibition increases GLS expression, thus increasing tumor dependency on glutamine as a potential resistance mechanism to mTOR inhibition [40, 41]. Although investigating the resistance mechanisms to mTOR inhibition was beyond the scope of this study, our data are consistent with a potential antitumor effect by telaglenastat in mTOR inhibitor-resistant tumors.

The rationale to combine cabozantinib with telaglenastat was based in part on observations that cabozantinib decreases glucose uptake measured via FDG-PET (coinciding with decreased expression of GLUT1/3) in thyroid cancer cells [24] and in animal models of colorectal cancer [25]. Other tyrosine kinase inhibitors, including apatinib and sunitinib, have also been associated with decreases in glycolysis, both in preclinical and clinical studies; metastatic RCC patients receiving sunitinib showed metabolic responses by FDG-PET as early as 2 weeks posttreatment [23]. Our results show that cabozantinb plus telaglenastat synergized to inhibit RCC cell proliferation in vitro and combined effectively to reduce both glucose and glutamine consumption, lower OCR, and suppress signal transduction pathways downstream of VEGFR, MET and AXL [59]. These three receptor tyrosine kinases signal via PI3K/AKT/mTOR and RAS/RAF/MEK/ERK [25], and are known, in part, to control the metabolism of glucose [5]. This phenomenon is consistent with findings that other receptor tyrosine kinases, such as EGFR [60], FGFR [61], FLT3 [62], AXL [63], PDGFR [64], IGF1R [65], ROS [66], and MET [67], have been shown to regulate glucose metabolism. We observed that the combination of telaglenastat plus cabozantinib in RCC tumor-bearing mice was well tolerated and led to enhanced anti-tumor activity. From our *in vitro* experiments, we surmise that the enhanced anti-tumor activity of cabozantinib plus telaglenastat is due to the dual inhibition of glucose and glutamine, but we cannot rule out other complementary mechanisms at play. For example, it is well established that treatment with TKIs such as cabozantinib lead to decreased tumor vascularity, which can further decrease the availability of oxygen and glucose, hence exacerbating the need for alternative fuels, such as glutamine. Under these conditions, the combination of anti-angiogenic therapies with telaglenastat is hypothesized to have a profound anti-tumor effect, as was empirically observed in this study. More work will be needed to fully characterize this *in vivo* mechanism of drug synergy.

## Conclusion

Recently, a number of new therapies have been approved for the treatment of advanced/metastatic RCC. These treatments generally fall into two mechanisms of action, checkpoint inhibitors and TKIs. However, given that current standard of care therapies are not curative in the advanced/metastatic setting for the vast majority of patients, there continues to be a high unmet need for therapies with new mechanisms of action [68]. RCC is a very metabolically active tumor that is highly reliant on glutamine and glutaminolysis for growth and proliferation. Targeting glutamine metabolism has been previously explored with other allosteric GLS inhibitors, such as BPTES and compound 968; however, these compounds lack the potency and bioavailability to be evaluated in clinical settings [69, 70]. Telaglenastat is a highly potent and selective, orally bioavailable GLS inhibitor with anti-proliferative activity in ccRCC and pRCC tumor-derived cell lines. The on-target inhibitory effect on GLS is supported by telaglenastat’s suppression of glutamate and glutamate-dependent metabolic products.

Dual inhibition of glutamine and glucose metabolism represents a promising therapeutic strategy for this highly metabolic tumor. Given that there are no approved agents that directly inhibit glucose metabolism, indirectly targeting glycolysis with signal transduction inhibitors in combination with glutaminase inhibition represents an attractive therapeutic strategy for RCC. Our work builds on prior studies that have demonstrated enhanced glucose utilization and glutamine dependency in RCC. In our study, we showed that combinations of telaglenastat with everolimus or cabozantinib lead to synergistic antiproliferative effects in vitro and teleglenastat enhanced anti-tumor effects of everolimus, cabozantinib, sunitinib, or axitinib in vivo. Collectively, our findings support targeting of key metabolic pathways, namely glutaminolysis and glycolysis, as a novel therapeutic strategy for RCC.

As with any preclinical study, whether these findings will translate to the clinical setting can only be determined in a clinical trial. The findings presented herein were relevant to ccRCC, but very few papillary or other histological types were studied, so generalization to other subtypes is currently unknown.

These preclinical data supported the initiation of ENTRATA, a phase 2, double-blind, randomized, placebo-controlled trial of telaglenastat plus everolimus in patients with RCC, which showed encouraging efficacy and safety data [71]. Data within also supported CANTATA, a double-blind, randomized, placebo-controlled trial of telaglenastat plus cabozantinib in patients with advanced/metastatic clear cell RCC. Findings from the clinical studies will be reported in another publication.

## Supporting information

S1 Table Cell line sensitivity to telaglenastat treatment or glutamine withdrawal. (PDF)

S1 Fig Dose response curves to telaglenastat treatment in ccRCC cell lines. The dashed lines indicate the relative CellTiter-Glo signal at the time of telaglenastat addition. EC_50_ values for each cell line are noted.
(PDF)

S2 Fig Dose response curves to telaglenastat treatment in pRCC, rhabdoid, and transitional kidney cancer cell lines. The dashed line indicates the relative CellTiter-Glo signal at the time of telaglenastat addition. EC_50_ values and histology for each line are noted.
(PDF)

S3 Fig GLS expression is enriched in papillary and clear cell RCC. GLS expression across 32 tumor types in the Cancer Genome Atlas Database (TCGA; https://www.cbioportal.org).
(PDF)

S4 Fig Expression of GLS in tumor vs. normal kidney. mRNA levels were obtained from Compendia Bioscience™ Translational Bioinformatics Services (Life Technologies, Ann Arbor, MI). mRNA expression levels are plotted as the log2 RNA normalized values. Whiskers span the 5th to 95th percentile with data outside this range shown as individual data points. Statistics were performed using Mann-Whitney t test to generate P values: *P ≤ 0.05, **** *P* ≤ 0.0001, ns (not significant).
(PDF)

S5 Fig Synergistic anti-proliferative activity of telaglenastat and everolimus in TUHR10TKB cells. Viability of TUHR10TKB cells treated with telaglenastat, everolimus, or a combination of both inhibitors for 72 hours. All experiments performed in triplicate or quadruplicate. Error bars represent standard deviations.
(PDF)

S6 Fig Telaglenastat plus everolimus decreases ECAR and OCR in RCC cell lines. Cells were treated with 1 μM telaglenastat and 100 nM everolimus for 24 hours prior to measurement. Determination of extracellular acidification rate and oxygen consumption rate using the Seahorse Metabolic Analyzer. Statistical significance was determined using RM 1-way ANOVA with Dunnett’s test for multiple comparisons: *P < 0.05; **P < 0.01; ***P < 0.001; ****P < 0.0001; ns = nonsignificant.
(PDF)

S7 Fig Body weights of mice implanted with Caki-1 RCC cells and treated with vehicle, telaglenastat (200 mg/kg, dosed orally BID), or (A) everolimus (1 mg/kg, dosed orally QD), (B) cabozantinib (1 mg/kg dosed orally QD), (C) sunitinib (20 mg/kg dosed orally QD), or (D) axitinib (25 mg/kg dosed orally QD), or combinations of telaglenast with each.
(PDF)

S8 Fig ECAR and OCR curves for telaglenastat combination studies in RCC cell lines analyzed in the seahorse metabolic analyzer. (A) Telaglenastat +/- everolimus in ACHN cells; (B) Telaglenastat +/- cabozantinib in Caki-1cells; (C) Telaglenastat + everolimus in RG2 cells; (D) MF cells; (E) FG2; (F) KMRC-1 cells, (G) KMRC-20 cells; (H) RCZ cells.
(PDF)

S1 Raw images (PDF)
