# Supplementary material for: The glutaminase inhibitor telaglenastat enhances the antitumor activity of signal transduction inhibitors everolimus and cabozantinib in models of renal cell carcinoma
Source: PLoS One. 2021 Nov 3;16(11):e0259241. doi: 10.1371/journal.pone.0259241 (PMC8565744; doi:10.1371/journal.pone.0259241)
Supplement: S1 Table — (PDF) [file pone.0259241.s001.pdf]

Table S1. Cell line sensitivity to telaglenastat treatement or glutamine withdrawal

Histology				Teleglenasat Dose Response		Response to telaglenastat (1 μM)						Response to Glutamine Withdrawal (1 μM)					
Ref [1]	Ref [2]	Ref [3]	Cellosaurus	Cell line	EC50 (nM)	Cell line	Exp. 1	Exp. 2	Exp. 3	mean	SEM	Cell line	Exp. 1	Exp. 2	Exp. 3	mean	SEM
cc	ND	cc	NA	TUHR10TKB	3	TUHR10TKB	-88	-91	-85	-88	2	TUHR10TKB	-77	-87	-70	-78	5
cc	cc	cc	NA	A-704	17	A-704	-72	-64	-61	-66	3	A-704	-56	-35	-34	-42	7
cc	cc	cc	NA	OS-RC-2	<0.1	OS-RC-2	-70	-59	-65	-65	3	OS-RC-2	-28	-35	-41	-34	4
cc	ND	cc	NA	VMRC-RCZ	3	VMRC-RCZ	-56	-67	-65	-63	3	VMRC-RCZ	-64	-49	-34	-49	9
cc	cc	cc	NA	786-0	13	786-0	-57	-69	-61	-62	4	786-0	0	-16	-9	-8	5
cc	ND	cc	cc	RCC-JW	7	RCC-JW	-30	-76	-71	-59	15	RCC-JW	-20	-19	-18	-19	1
cc	ND	cc	NA	VMRC-RCW	65	VMRC-RCW	-58	-49	-47	-52	3	VMRC-RCW	-25	-15	-14	-18	4
cc	ND	cc	cc	RCC-JF	35	RCC-JF	-46	-57	-47	-50	4	RCC-JF	-47	-42	-34	-41	4
cc	ND	cc	cc	KMRC-20	9	KMRC-20	-44	-43	-48	-45	2	KMRC-20	-23	-30	-24	-25	2
cc	ND	cc	cc	RCC-MF	0.3	RCC-MF	-43	-38	-43	-41	2	RCC-MF	-55	-55	-68	-59	4
cc	cc	cc	cc	Caki-1	170	Caki-1	-51	-42	-30	-41	6	Caki-1	-58	-48	-49	-52	3
cc	ND	cc	NA	RCC-10RGB	92	RCC-10RGB	-29	-22	-57	-36	11	RCC-10RGB	-7	15	-42	-12	17
cc	ND	cc	cc	KMRC-1	61	KMRC-1	-29	-57	-4	-30	15	KMRC-1	-31	-56	-55	-47	8
cc	cc	cc	NA	A498	550	A498	-25	-37	-22	-28	5	A498	-14	-33	-2	-16	9
cc	cc	cc	NA	769-P	30	769-P	-41	9	-29	-20	15	769-P	-59	-36	-57	-51	7
cc	ND	ND	cc	RCC-GH	98	RCC-GH	4	-32	-21	-16	11	RCC-GH	-35	-36	-37	-36	1
cc	ND	cc	cc	KMRC-3	0.6	KMRC-3	3	-9	-29	-12	9	KMRC-3	-28	-31	-14	-24	5
cc	cc	ND	cc	RCC-FG1	120	RCC-FG1	-9	13	-23	-6	10	RCC-FG1	-19	-8	-12	-13	3
rhab	ND	ND	rhab	WT-CLS1	43	WT-CLS1	-27	11	22	2	15	WT-CLS1	17	24	49	30	10
pap	ND	pap	NA	Cal-54	230	Cal-54	10	9	4	7	2	Cal-54	11	8	7	9	1
pap	pap	cc	pap	Caki-2	46	Caki-2	16	11	2	10	4	Caki-2	-19	-25	-32	-25	4
rhab	ND	ND	rhab	JMU-RTK-2	>1000	JMU-RTK-2	11	28	-7	11	10	JMU-RTK-2	51	79	51	60	9
pap	pap/cc	pap	pap	ACHN	230	ACHN	17	36	1	18	10	ACHN	-6	4	-14	-5	5
cc	cc	cc	cc	RCC-ER	>1000	RCC-ER	72	43	26	47	13	RCC-ER	39	24	37	33	5
cc	ND	cc	cc	RCC-FG2	>1000	RCC-FG2	60	62	67	63	2	RCC-FG2	0	-19	3	-5	7
rhab	ND	ND	rhab	G401	>1000	G401	76	80	no data	78	2	G401	37	42	no data	40	3
trans	ND	cc	trans	BFTC-909	>1000	BFTC-909	81	90	no data	86	5	BFTC-909	15	11	no data	13	2

cc, clear cell; Exp., experiment; NA, not available; ND; no data; rhab, rhabdoid; SEM, standard error of the mean; trans, transitional cell carcinoma

Cell death (negative number) or cell growth (positive number) following 72 hour telaglenastat treatment. Cell line histology was assigned based on previously published reports and cellosaurus (https://web.expasy.org/cellosaurus/). Cell lines are arranged in the order they appear in Figure 1A.

[1] Emberley et al. [2] Brodaczewska KK et al. Mol Cancer. 2016;15:83; [3] Sinha R et al. Nat Commun. 2017;8:15165.
