# Supplementary material for: The glutaminase inhibitor telaglenastat enhances the antitumor activity of signal transduction inhibitors everolimus and cabozantinib in models of renal cell carcinoma
Source: PLoS One. 2021 Nov 3;16(11):e0259241. doi: 10.1371/journal.pone.0259241 (PMC8565744; doi:10.1371/journal.pone.0259241)
Supplement: S1 Fig — The dashed lines indicate the relative CellTiter-Glo signal at the time of telaglenastat addition. EC50 values for each cell line are noted. (PDF) [file pone.0259241.s002.pdf]

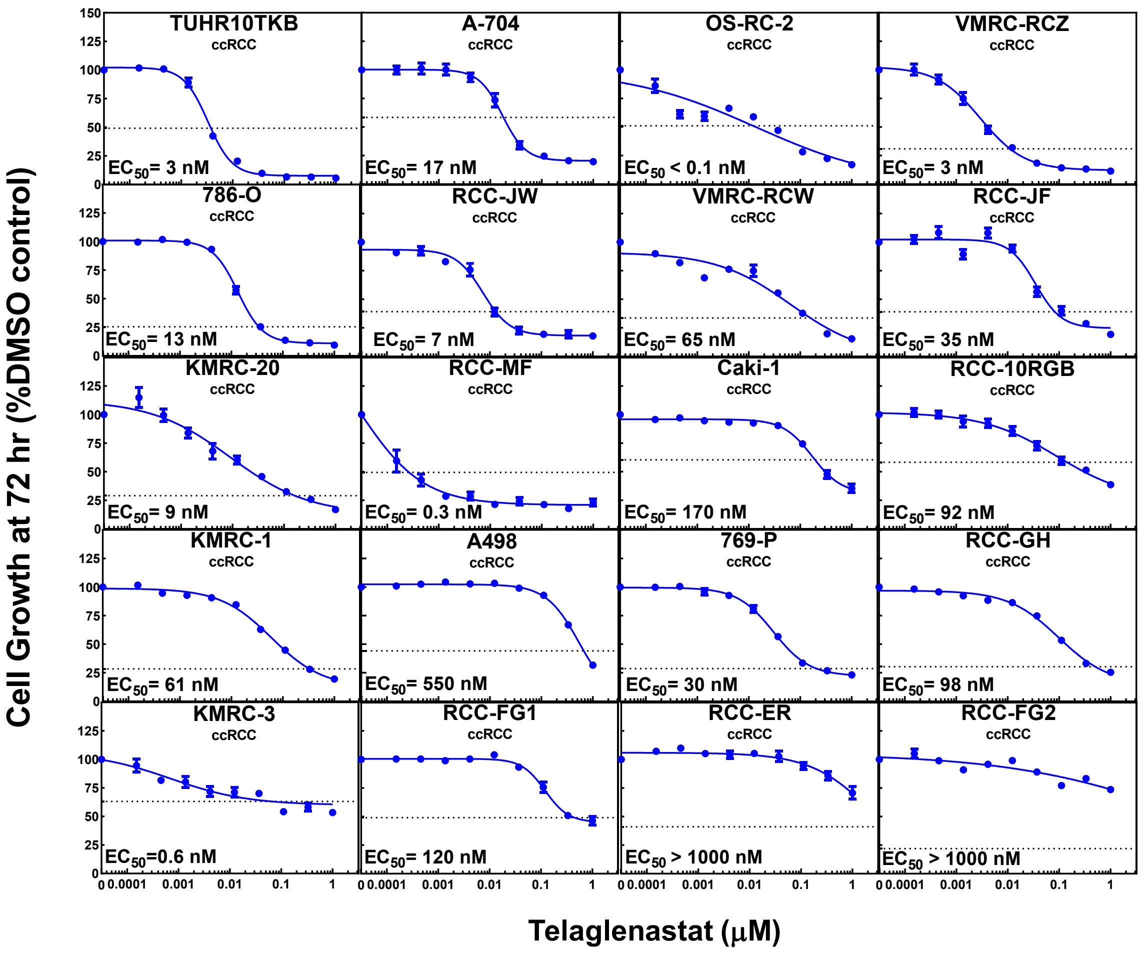

Figure S1. Dose response curves to telaglenastat treatment in ccRCC cell lines. The dashed lines indicate the relative CellTiter-Glo signal at the time of telaglenastat addition. EC_{50} values for each cell line are noted.
