# Supplementary material for: The glutaminase inhibitor telaglenastat enhances the antitumor activity of signal transduction inhibitors everolimus and cabozantinib in models of renal cell carcinoma
Source: PLoS One. 2021 Nov 3;16(11):e0259241. doi: 10.1371/journal.pone.0259241 (PMC8565744; doi:10.1371/journal.pone.0259241)
Supplement: S2 Fig — The dashed line indicates the relative CellTiter-Glo signal at the time of telaglenastat addition. EC50 values and histology for each line are noted. (PDF) [file pone.0259241.s003.pdf]

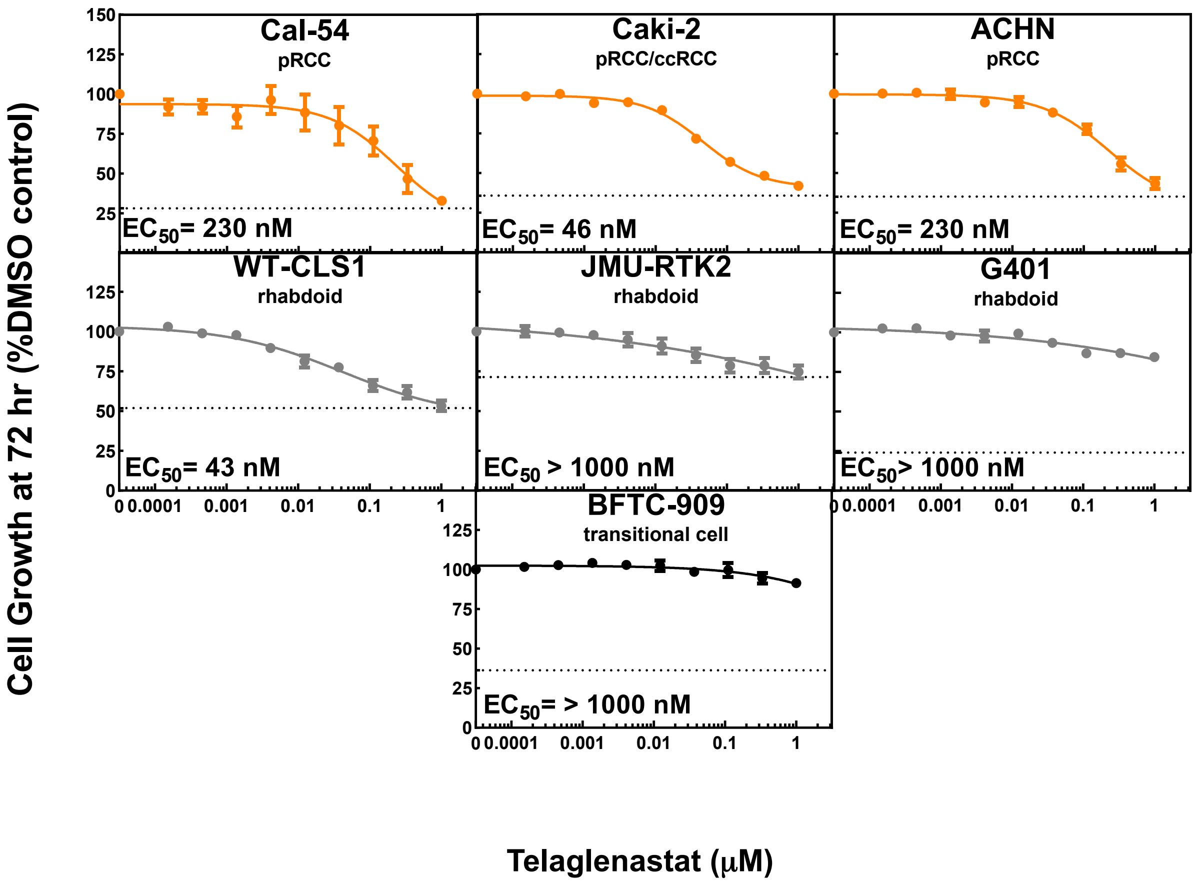

Figure S2. Dose response curves to telaglenastat treatment in pRCC, rhabdoid, and transitional kidney cancer cell lines. The dashed line indicates the relative CellTiter-Glo signal at the time of telaglenastat addition. EC_{50} values and histology for each line are noted.
