# Supplementary material for: The glutaminase inhibitor telaglenastat enhances the antitumor activity of signal transduction inhibitors everolimus and cabozantinib in models of renal cell carcinoma
Source: PLoS One. 2021 Nov 3;16(11):e0259241. doi: 10.1371/journal.pone.0259241 (PMC8565744; doi:10.1371/journal.pone.0259241)
Supplement: S3 Fig — GLS expression across 32 tumor types in the Cancer Genome Atlas Database (TCGA; https://www.cbioportal.org). (PDF) [file pone.0259241.s004.pdf]

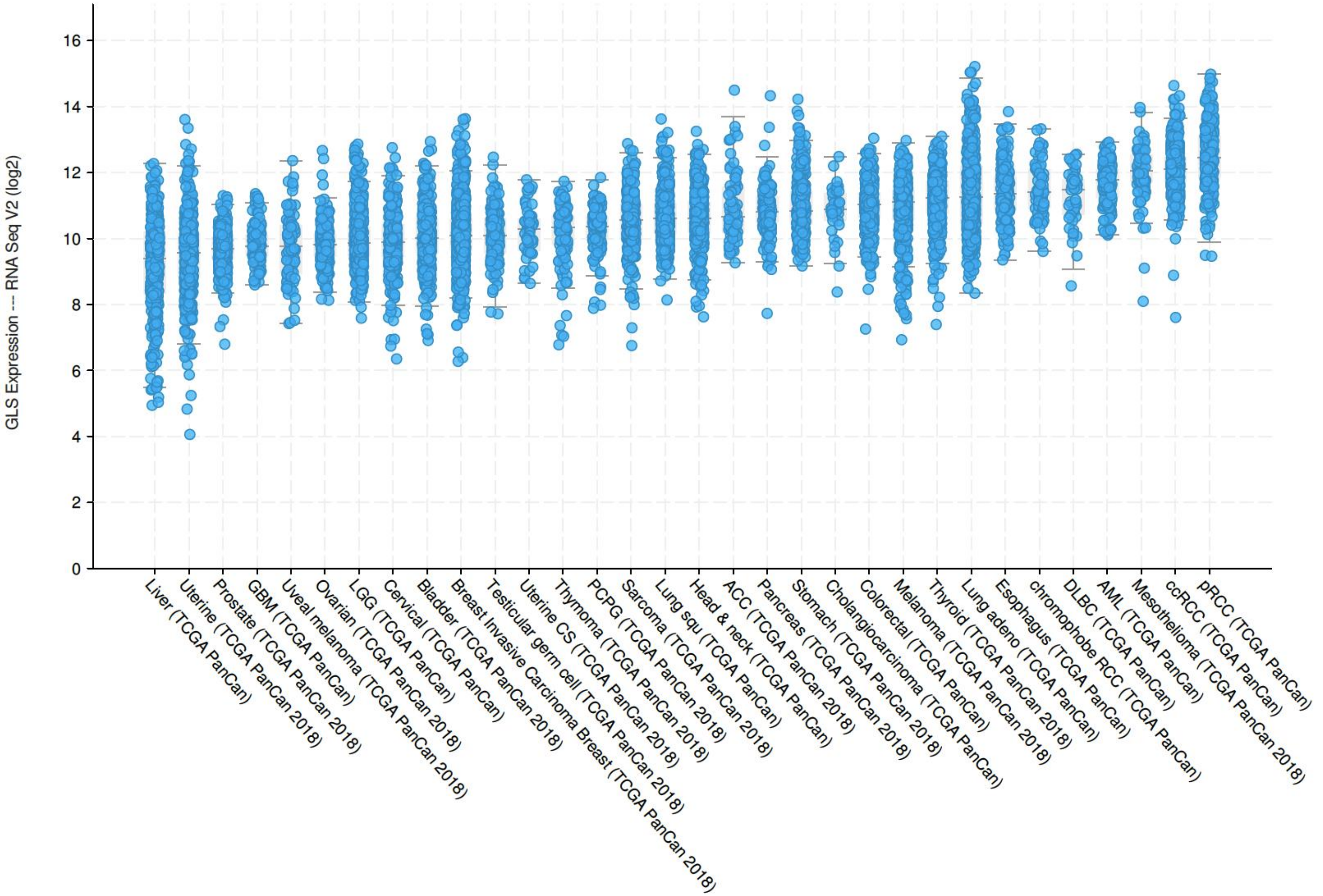

Figure S3. GLS Expression is Enriched in Papillary and Clear Cell RCC. GLS expression across 32 tumor types in the Cancer Genome Atlas Database (TCGA; https://www.cbioportal.org).
