# Supplementary material for: The glutaminase inhibitor telaglenastat enhances the antitumor activity of signal transduction inhibitors everolimus and cabozantinib in models of renal cell carcinoma
Source: PLoS One. 2021 Nov 3;16(11):e0259241. doi: 10.1371/journal.pone.0259241 (PMC8565744; doi:10.1371/journal.pone.0259241)
Supplement: S4 Fig — mRNA levels were obtained from Compendia Bioscience™ Translational Bioinformatics Services (Life Technologies, Ann Arbor, MI). mRNA expression levels are plotted as the log2 RNA normalized values. Whiskers span the 5th to 95th percentile with data outside this range shown as individual data points. Statistics were performed using Mann-Whitney t test to generate P values: *P ≤ 0.05, **** P ≤ 0.0001, ns (not significant). (PDF) [file pone.0259241.s005.pdf]

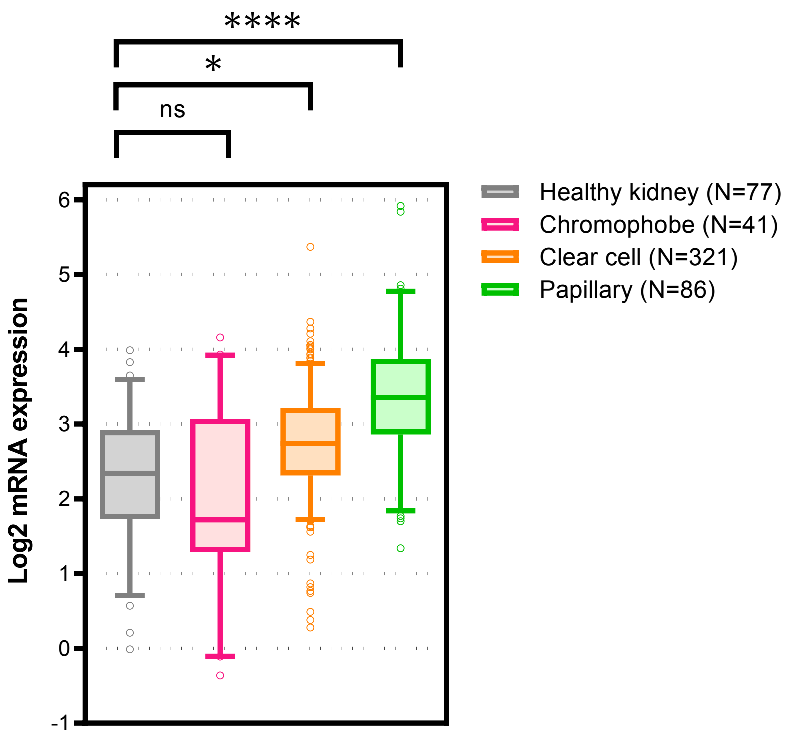

Figure S4. Expression of GLS in tumor vs. normal kidney. mRNA levels were obtained from Compendia Bioscience™ Translational Bioinformatics Services (Life Technologies, Ann Arbor, MI). mRNA expression levels are plotted as the log2 RNA normalized values. Whiskers span the 5th to 95th percentile with data outside this range shown as individual data points. Statistics were performed using Mann-Whitney t test to generate P values: *$P \leq 0.05$, **** $P \leq 0.0001$, ns (not significant).
