# Supplementary material for: The glutaminase inhibitor telaglenastat enhances the antitumor activity of signal transduction inhibitors everolimus and cabozantinib in models of renal cell carcinoma
Source: PLoS One. 2021 Nov 3;16(11):e0259241. doi: 10.1371/journal.pone.0259241 (PMC8565744; doi:10.1371/journal.pone.0259241)
Supplement: S5 Fig — Viability of TUHR10TKB cells treated with telaglenastat, everolimus, or a combination of both inhibitors for 72 hours. All experiments performed in triplicate or quadruplicate. Error bars represent standard deviations. (PDF) [file pone.0259241.s006.pdf]

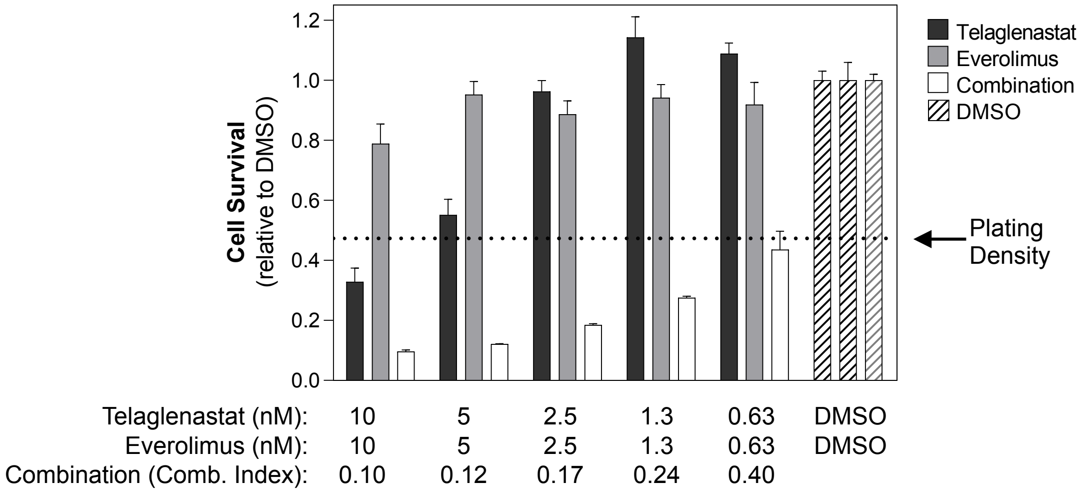

Figure S5. Synergistic anti-proliferative activity of telaglenastat and everolimus in TUHR10TKB cells. Viability of TUHR10TKB cells treated with telaglenastat, everolimus, or a combination of both inhibitors for 72 hours. All experiments performed in triplicate or quadruplicate. Error bars represent standard deviations.
