# Supplementary material for: The glutaminase inhibitor telaglenastat enhances the antitumor activity of signal transduction inhibitors everolimus and cabozantinib in models of renal cell carcinoma
Source: PLoS One. 2021 Nov 3;16(11):e0259241. doi: 10.1371/journal.pone.0259241 (PMC8565744; doi:10.1371/journal.pone.0259241)
Supplement: S6 Fig — Cells were treated with 1 μM telaglenastat and 100 nM everolimus for 24 hours prior to measurement. Determination of extracellular acidification rate and oxygen consumption rate using the Seahorse Metabolic Analyzer. Statistical significance was determined using RM 1-way ANOVA with Dunnett’s test for multiple comparisons: *P < 0.05; **P < 0.01; ***P < 0.001; ****P < 0.0001; ns = nonsignificant. (PDF) [file pone.0259241.s007.pdf]

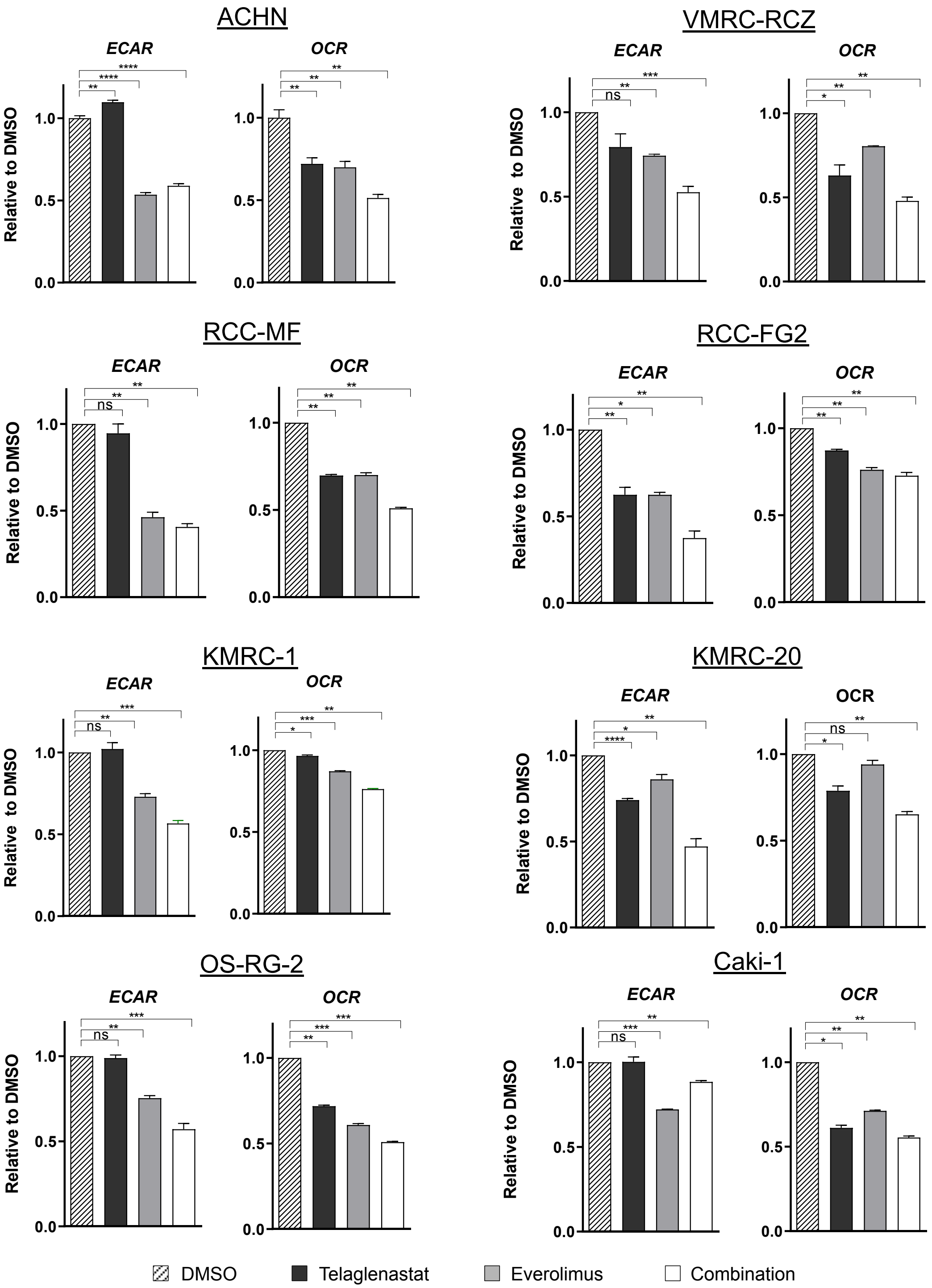

Figure S6. Telaglenastat plus everolimus decreases ECAR and OCR in RCC cell lines. Cells were treated with 1 µM telaglenastat and 100 nM everolimus for 24 hours prior to measurement. Determination of extracellular acidification rate and oxygen consumption rate using the Seahorse Metabolic Analyzer. Statistical significance was determined using RM 1-way ANOVA with Dunnett's test for multiple comparisons: *$P < 0.05$; **$P < 0.01$; ***$P < 0.001$; ****$P < 0.0001$; ns = nonsignificant.
