# Supplementary material for: The glutaminase inhibitor telaglenastat enhances the antitumor activity of signal transduction inhibitors everolimus and cabozantinib in models of renal cell carcinoma
Source: PLoS One. 2021 Nov 3;16(11):e0259241. doi: 10.1371/journal.pone.0259241 (PMC8565744; doi:10.1371/journal.pone.0259241)
Supplement: S7 Fig — Body weights of mice implanted with Caki-1 RCC cells and treated with vehicle, telaglenastat (200 mg/kg, dosed orally BID), or (A) everolimus (1 mg/kg, dosed orally QD), (B) cabozantinib (1 mg/kg dosed orally QD), (C) sunitinib (20 mg/kg dosed orally QD), or (D) axitinib (25 mg/kg dosed orally QD), or combinations of telaglenast with each. (PDF) [file pone.0259241.s008.pdf]

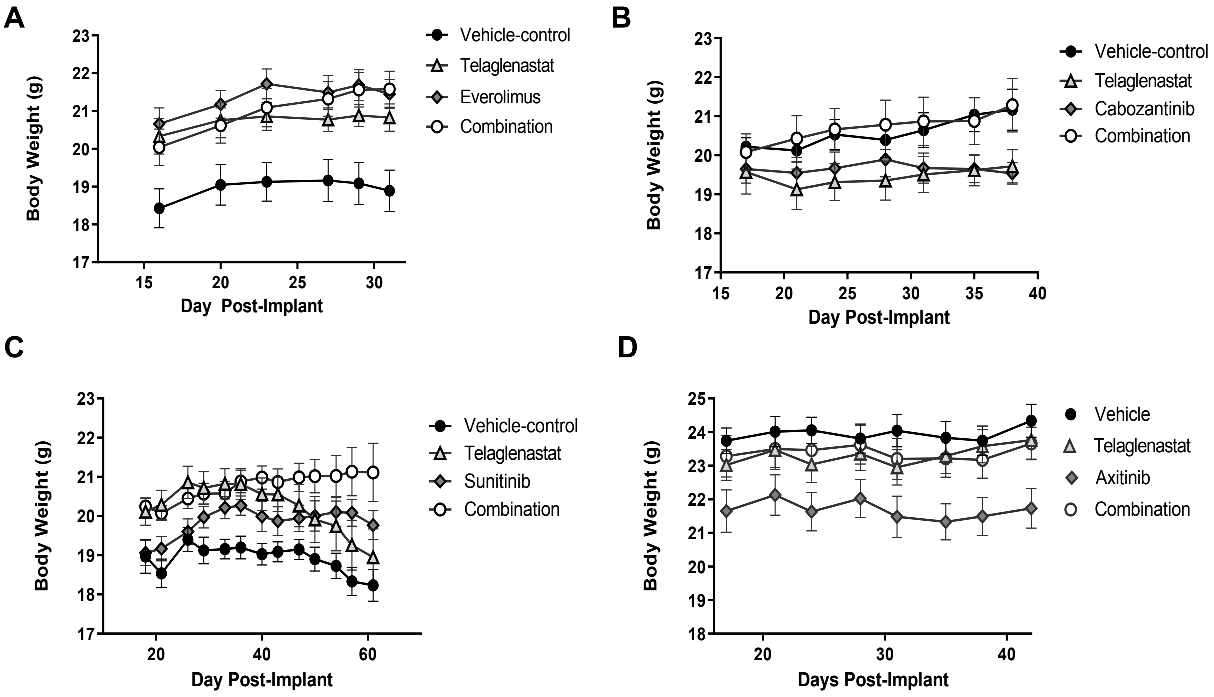

Figure S7. Body weights of mice implanted with Caki-1 RCC cells and treated with vehicle, telaglenastat (200 mg/kg, dosed orally BID), or (A) everolimus (1 mg/kg, dosed orally QD), (B) cabozantinib (1 mg/kg dosed orally QD), (C) sunitinib (20 mg/kg dosed orally QD), or (D) axitinib (25 mg/kg dosed orally QD), or combinations of telaglenast with each.
