# Supplementary material for: The glutaminase inhibitor telaglenastat enhances the antitumor activity of signal transduction inhibitors everolimus and cabozantinib in models of renal cell carcinoma
Source: PLoS One. 2021 Nov 3;16(11):e0259241. doi: 10.1371/journal.pone.0259241 (PMC8565744; doi:10.1371/journal.pone.0259241)
Supplement: S8 Fig — (A) Telaglenastat +/- everolimus in ACHN cells; (B) Telaglenastat +/- cabozantinib in Caki-1cells; (C) Telaglenastat + everolimus in RG2 cells; (D) MF cells; (E) FG2; (F) KMRC-1 cells, (G) KMRC-20 cells; (H) RCZ cells. (PDF) [file pone.0259241.s009.pdf]

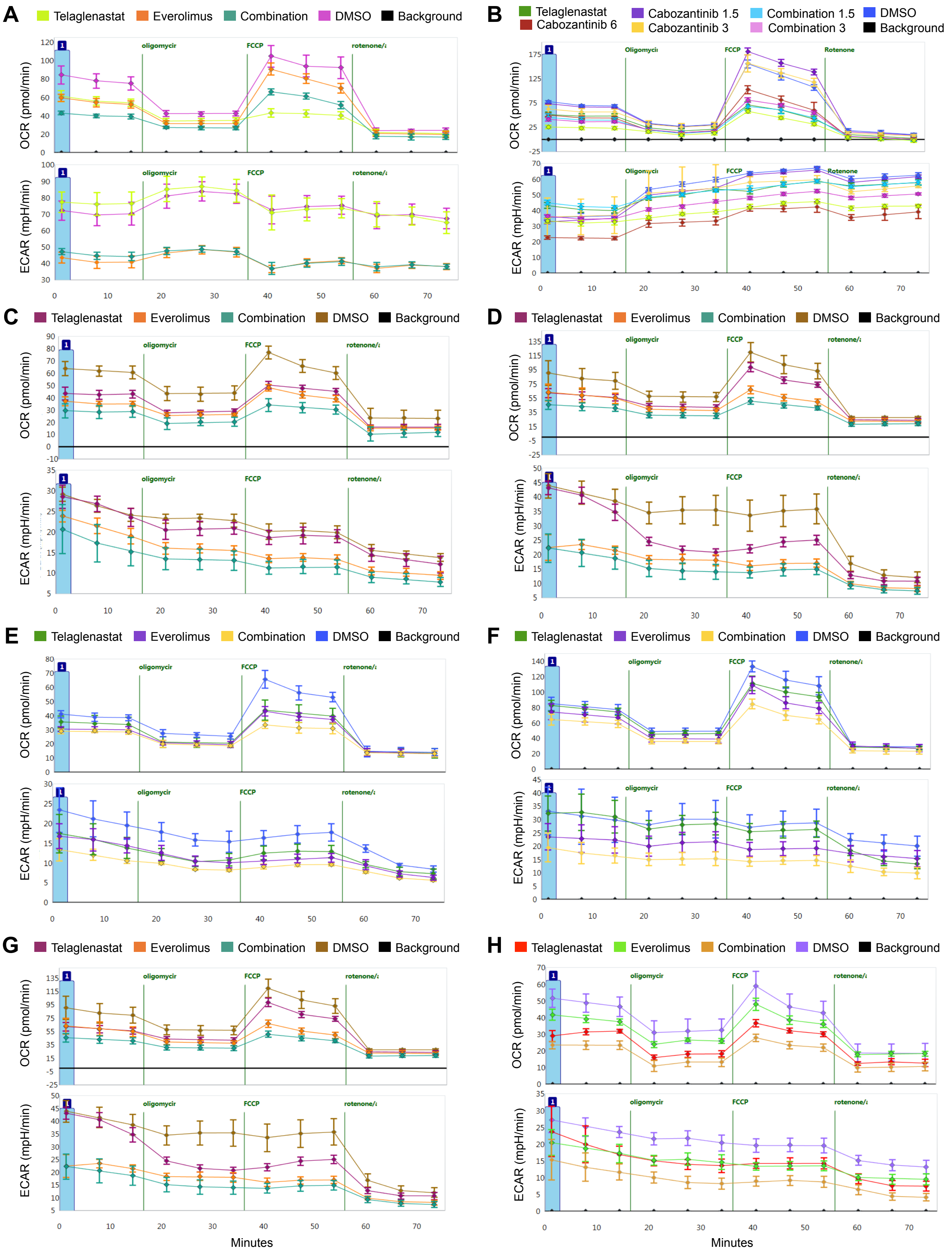

ECAR and OCR curves for telaglenastat combination studies in RCC cell lines analyzed in the Seahorse Metabolic Analyzer. (A) Telaglenastat +/- everolimus in ACHN cells; (B) Telaglenastat +/- cabozantinib in Caki-1cells; (C) Telaglenastat + everolimus in RG2 cells; (D) MF cells; (E) FG2; (F) KMRC-1 cells, (G) KMRC-20 cells; (H) RCZ cells.
