# Supplementary material for: The glutaminase inhibitor telaglenastat enhances the antitumor activity of signal transduction inhibitors everolimus and cabozantinib in models of renal cell carcinoma
Source: PLoS One. 2021 Nov 3;16(11):e0259241. doi: 10.1371/journal.pone.0259241 (PMC8565744; doi:10.1371/journal.pone.0259241)
Supplement: S1 Raw images — (PDF) [file pone.0259241.s010.pdf]

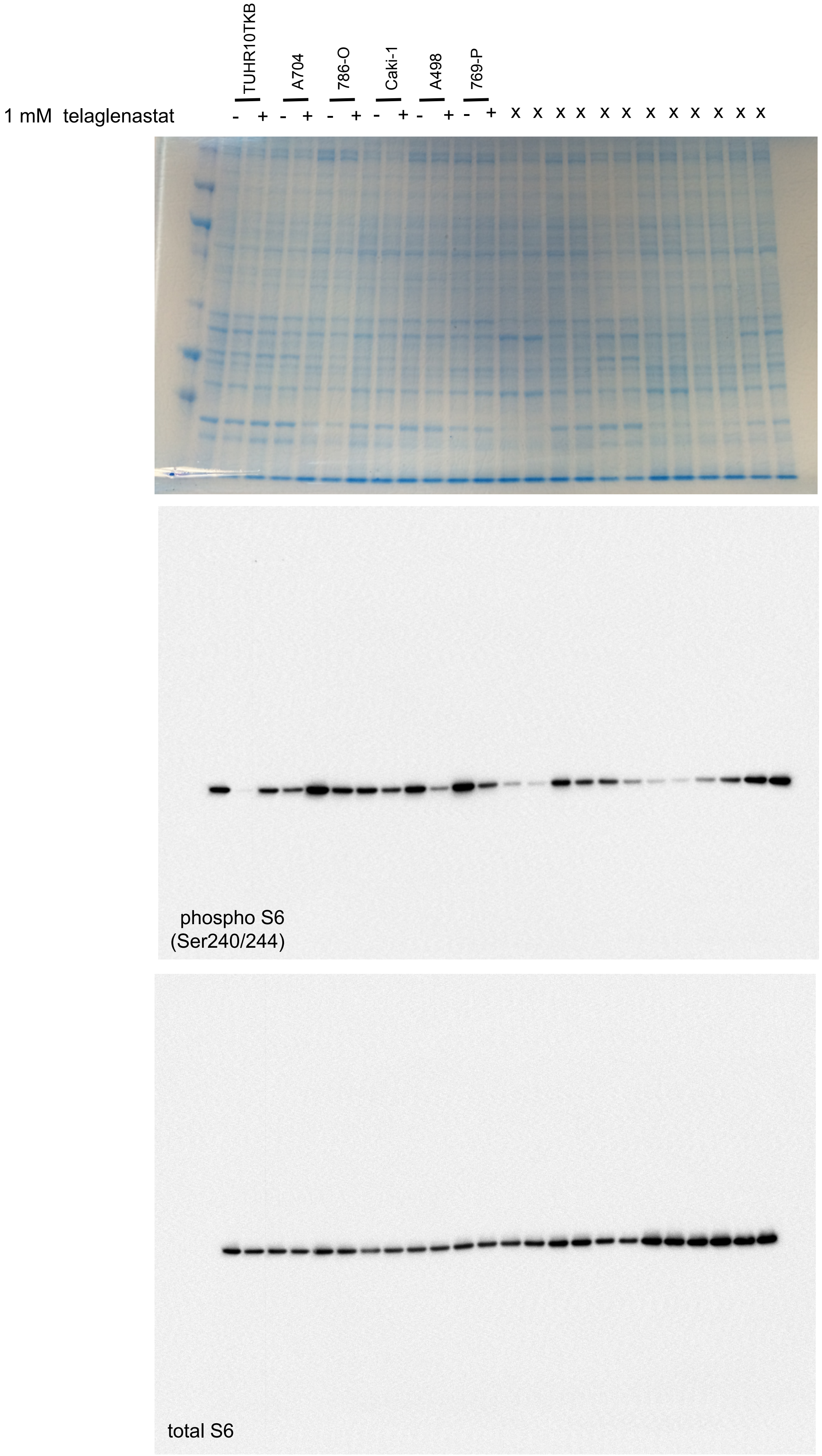

Coomassie-stained gel and anti-phospho S6 and anti-S6 western blots from Fig. 2A in the main text.

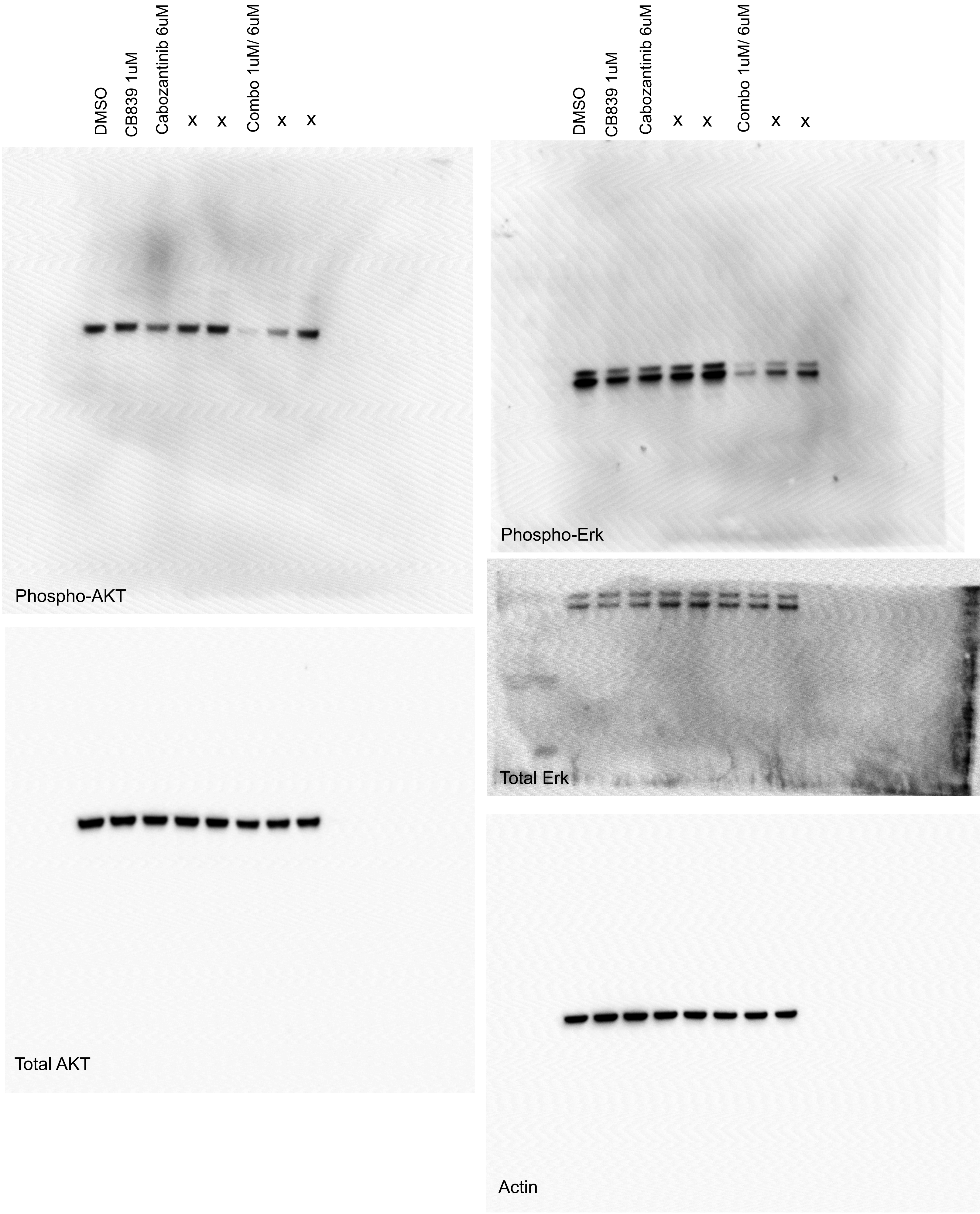

Anti-phospho AKT, anti-AKT, anti-phospho ERK, anti-ERK, and anti-actin western blots from Fig. 4B in the main text.

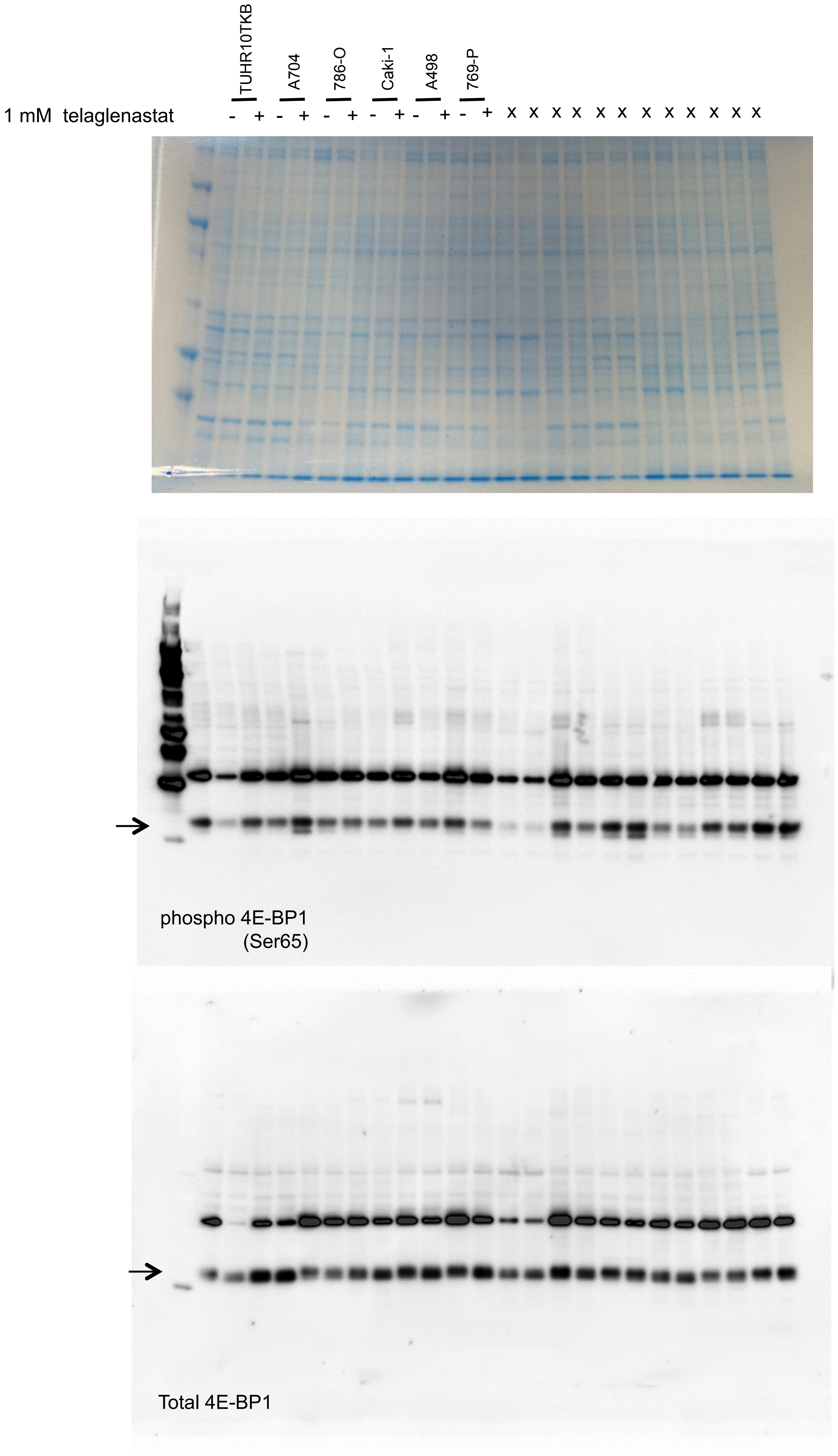

Coomassie-stained gel and anti-phospho 4E-BP1 and anti-4E-BP1 western blots from Fig. S5 in the supporting information.
